# Interaction of the Psychiatric Risk Gene *Cacna1c* With Post-weaning Social Isolation or Environmental Enrichment Does Not Affect Brain Mitochondrial Bioenergetics in Rats

**DOI:** 10.3389/fncel.2019.00483

**Published:** 2019-10-25

**Authors:** Susanne Michels, Amalia M. Dolga, Moria D. Braun, Theresa M. Kisko, A. Özge Sungur, Stephanie H. Witt, Marcella Rietschel, Astrid Dempfle, Markus Wöhr, Rainer K. W. Schwarting, Carsten Culmsee

**Affiliations:** ^1^Institute of Pharmacology and Clinical Pharmacy, University of Marburg, Marburg, Germany; ^2^Center for Mind, Brain and Behavior, University of Marburg, Marburg, Germany; ^3^Department of Molecular Pharmacology, Faculty of Science and Engineering, Groningen Research Institute of Pharmacy (GRIP), University of Groningen, Groningen, Netherlands; ^4^Department of Experimental and Biological Psychology, University of Marburg, Marburg, Germany; ^5^Department of Genetic Epidemiology in Psychiatry, Central Institute of Mental Health, Mannheim, Germany; ^6^Institute of Medical Informatics and Statistics, Kiel University, Kiel, Germany

**Keywords:** brain bioenergetics, Ca_*V*_1.2, gene x environment interaction, isolated mitochondria, neuropsychiatric disorders

## Abstract

The pathophysiology of neuropsychiatric disorders involves complex interactions between genetic and environmental risk factors. Confirmed by several genome-wide association studies, *Cacna1c* represents one of the most robustly replicated psychiatric risk genes. Besides genetic predispositions, environmental stress such as childhood maltreatment also contributes to enhanced disease vulnerability. Both, *Cacna1c* gene variants and stressful life events are associated with morphological alterations in the prefrontal cortex and the hippocampus. Emerging evidence suggests impaired mitochondrial bioenergetics as a possible underlying mechanism of these regional brain abnormalities. In the present study, we simulated the interaction of psychiatric disease-relevant genetic and environmental factors in rodents to investigate their potential effect on brain mitochondrial function using a constitutive heterozygous *Cacna1c* rat model in combination with a four-week exposure to either post-weaning social isolation, standard housing, or social and physical environmental enrichment. Mitochondria were isolated from the prefrontal cortex and the hippocampus to evaluate their bioenergetics, membrane potential, reactive oxygen species production, and respiratory chain complex protein levels. None of these parameters were considerably affected in this particular gene-environment setting. These negative results were very robust in all tested conditions demonstrating that *Cacna1c* depletion did not significantly translate into altered bioenergetic characteristics. Thus, further investigations are required to determine the disease-related effects on brain mitochondria.

## Introduction

Neuropsychiatric disorders such as major depression, bipolar disorder, schizophrenia, and autism are highly prevalent, multifaceted, and heterogeneous diseases with shared symptomatic features (Adam, 2013). Despite being among the leading causes of disability worldwide, their underlying pathophysiology still remains poorly understood (Kessler et al., 2009). Established current neurobiological theories attempting to elucidate the pathophysiology of these complex psychiatric disorders focus on genetic and environmental risk factors, and their interactions (Keers and Uher, 2012).

Identified by genome-wide association studies, *Cacna1c* represents the most robustly replicated psychiatric risk gene in major depression, bipolar disorder, schizophrenia, and autism. *Cacna1c* encodes the α_1__*C*_ subunit of the major L-type calcium channel (LTCC) in the brain (Ca_*V*_1.2) (Bhat et al., 2012; Cross-Disorder Group of the Psychiatric Genomics Consortium, 2013). Several studies provide convincing evidence that the risk variant rs1006737 is associated with altered Ca_*V*_1.2 expression, impaired calcium signaling, as well as compromised structure and function of brain regions relevant for characteristic behavioral changes observed in psychiatric patients (Bhat et al., 2012; Erk et al., 2014; Paulus et al., 2014). Particularly affected brain regions include the prefrontal cortex (PFC) and the hippocampus (HC), which are essential for stress regulation, emotion, cognition, learning, and memory (Busatto, 2013). Supportive findings from transgenic animal models confirm the impact of the cross-disorder risk gene *Cacna1c* on PFC- and HC-dependent behaviors. For instance, constitutive *Cacna1c* haploinsufficiency leads to increased anxiety and antidepressant-like effects in mice and causes sex-specific deficits in social behavior and affective communication in juvenile as well as adult rats (Dao et al., 2010; Kabir et al., 2017; Kisko et al., 2018a, b; Redecker et al., 2019). Furthermore, embryonic deletion of *Cacna1c* in mouse forebrain glutamatergic neurons results in diminished synaptic plasticity, cognitive decline, and reduced sociability (Dedic et al., 2018).

Susceptibility to psychiatric illnesses is only partly genetic, with environmental stress such as childhood neglect and maltreatment representing an additional important contributing factor (Nanni et al., 2012). In line with the effects of *Cacna1c* gene variants, these adverse experiences in early-life are also linked to morphological brain alterations, in this case PFC and HC volume loss (Frodl et al., 2010; Opel et al., 2014). Physical and emotional neglect during childhood can be simulated in rodents by post-weaning social isolation, which induces behavioral phenotypes related to various neuropsychiatric disorders including social withdrawal and cognitive inflexibility (Seffer et al., 2015). Moreover, these prominent impairments due to juvenile social deprivation are accompanied by reduced PFC volume and decreased hippocampal synaptic plasticity (Fone and Porkess, 2008). On the contrary, social and physical environmental enrichment, mimicking positive and protective life experiences, promotes HC neurogenesis, improves learning and memory, reduces depression-related behavior, and has beneficial effects on affective communication through ultrasonic vocalizations in rats (Brenes et al., 2009, 2016).

Various epidemiologic studies indicate that a single risk factor is not sufficient for the development of a certain psychiatric disorder. These complex diseases rather result from multiple interdependent processes involving the interaction of different genetic and environmental factors (Keers and Uher, 2012). At the cellular level, the substantial impact of genetic and environmental risks as well as their interplay on neuroplasticity and behavior might be mediated by alterations in mitochondrial functioning, which have been frequently associated with psychiatric disorders (Manji et al., 2012). In mouse hippocampal HT22 cells, we recently found that siRNA-mediated knockdown of *Cacna1c* promotes mitochondrial resilience to oxidative stress, which reflects a common cellular response to environmental stress (Michels et al., 2018a, b). Mitochondria are crucial for cellular energy metabolism and play an important role in regulating calcium homeostasis, redox balance, synaptic plasticity, and cell viability, thereby influencing neural circuits that control high-order functions of the brain such as mood, cognition, and social behavior (Klinedinst and Regenold, 2015). In this context, emerging evidence strongly suggests impaired mitochondrial bioenergetics as possible underlying mechanism of regional brain abnormalities in mood disorders by compromising energy-dependent processes such as neuronal plasticity and the brain’s ability to resist or adapt to environmental stressors (Sousa et al., 2014).

Based on these findings, we hypothesized that the interaction of defined psychiatric disease-relevant genetic and environmental factors possibly affects brain mitochondrial function. To this aim, we used a constitutive heterozygous *Cacna1c* rat model in combination with a 4-week exposure to either social isolation, standard housing, or social and physical environmental enrichment during the juvenile developmental period. Subsequently, mitochondria were isolated from PFC and HC, both representing particularly susceptible brain regions in neuropsychiatric disorders, to evaluate their bioenergetics, membrane potential, reactive oxygen species (ROS) production, and respiratory chain complex protein levels.

## Materials and Methods

### Animals and Breeding

Constitutive heterozygous *Cacna1c*^+/–^ rats were generated by SAGE Labs (now Horizon Discovery Ltd., Cambridge, United Kingdom) on a Sprague Dawley background via zinc-finger nucleases following a previously established protocol (Geurts et al., 2009). *Cacna1c*^+/–^ rats carry a 4 base pair (bp) deletion at 460649–460652 bp in the genomic sequence resulting in an early stop codon in exon 6. Homozygous *Cacna1c*^–/–^ rats are embryonically lethal. Genotyping was performed as reported before (Kisko et al., 2018a) using the following primers: 5′-GCTGCTGAGCCTTTTATTGG-3′ (*Cacna1c* Cel-1 F) and 5′-CCTCCTGGATAGCTGCTGAC-3′ (*Cacna1c* Cel-1 R).

As published previously, a heterozygous breeding protocol was used to obtain offspring from both genotypes (Kisko et al., 2018a). To this aim, Sprague Dawley females (Charles River, Sulzfeld, Germany) and male *Cacna1c*^+/–^ rats were paired for breeding. Sprague Dawley females were used because breeding efficacy is reduced in female *Cacna1c*^+/–^ rats. In order to avoid litter effects, only litters with both genotypes present were included in the experiments. Breeding was performed at the Faculty of Psychology, University of Marburg, Germany. Approximately 2 weeks after pairing for breeding, females were individually housed and inspected daily for pregnancy and delivery. The day of birth was considered as postnatal day (PND) 0. Rats were identified by paw tattoo, using non-toxic animal tattoo ink (Ketchum permanent tattoo inks green paste; Ketchum Manufacturing Inc., Brockville, Canada). The ink was inserted subcutaneously through a 30-gauge hypodermic needle tip into the center of the paw on PND 5 ± 1. Rats were housed under standard laboratory conditions (22 ± 2°C and 40–70% humidity) with free access to standard rodent chow and water.

All procedures were reported in compliance with the ARRIVE guidelines (Animal Research: Reporting *in vivo* Experiments) and were conducted in strict accordance with the National Institutes of Health Guidelines for the Care and Use of Laboratory Animals and the relevant local or national rules and regulations of Germany and were subject to prior authorization by the local government (MR 20/35 Nr. 19/2014; Tierschutzbehörde, Regierungspräsidium Gießen, Germany).

### Gene x Environment Study

**FIGURE 1:**
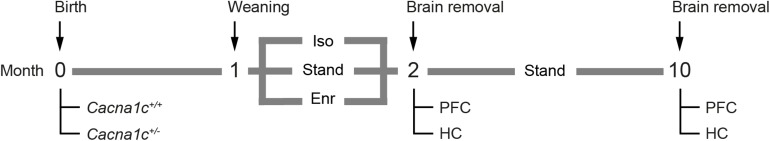
Experimental design. Shortly after birth, the genotypes of the rats (*Cacna1c*^+/+^ vs. *Cacna1c*^+/–^) were determined. With 3 weeks of age (PND 21), the rats were separated from their mothers and allocated to one of three experimental housing conditions, i.e., post-weaning social isolation (Iso), standard housing (Stand), and social and physical environmental enrichment (Enr), where they spend 4 weeks before their brains were removed (PND 50 ± 1) and the mitochondria were isolated from the prefrontal cortex (PFC) and the hippocampus (HC).

To study the effects of a gene x environment (GxE) interaction on brain mitochondrial bioenergetics, we applied a 2 × 3 design and exposed male *Cacna1c*^+/–^ rats and *Cacna1c*^+/+^ littermate controls to one of three experimental housing conditions for four consecutive weeks after weaning on PND 21, i.e., between PND 22 and 50 ± 1 (Figure 1). From each litter, six rats were included in the experiment whenever possible with a pair of *Cacna1c*^+/–^ and *Cacna1c*^+/+^ siblings each being randomly exposed to (A) post-weaning social isolation (Iso), (B) standard housing (Stand), or (C) social and physical environmental enrichment (Enr).

(A) Post-weaning social isolation (Iso) in a Makrolon type III cage (425 × 265 × 150 mm, plus high stainless-steel covers; Tecniplast Deutschland GmbH, Hohenpeißenberg, Germany), housed alone, applying a previously established protocol (Seffer et al., 2015).

(B) Standard housing (Stand) in a polycarbonate Makrolon Type IV cage (580 × 380 × 200 mm, plus high stainless-steel covers; Tecniplast Deutschland GmbH, Hohenpeißenberg, Germany), housed in groups of six rats, consistent with previously applied control conditions (Seffer et al., 2015).

(C) Social and physical environmental enrichment (Enr) in a large commercial rat cage (AniOne Remus, 104 × 59 × 107 cm; MultiFit Tiernahrungs GmbH, Krefeld, Germany) containing three wooden platforms connected by ramps, two metal food dispensers, and an assortment of cage accessories and places to hide, applying a modified protocol previously established (Brenes et al., 2016). The initial cage setup comprised six hiding places (2x Rodent Retreats red, Bio-Serv, Flemington, NJ, United States; 2× AniOne Grasnest Size L, MultiFit Tiernahrungs GmbH, Krefeld, Germany; 1× AniOne Grastunnel, MultiFit Tiernahrungs GmbH, Krefeld, Germany; 1× empty cardboard box, Dallmayr capsa, Munich, Germany), six wooden sticks for the animals to chew on, and two petri dishes with water in addition to the water bottle affixed to the cage wall. Twice a week, various accessories were either added to the cage (e.g., 2× wire feeding balls stuffed with paper tissue, Food-Ball Ø 12 cm, TRIXIE Heimtierbedarf GmbH, Tarp, Germany; 3× marbles; 2× empty toilet paper rolls) or exchanged, in the case of hiding places (1× wooden rat house, Jesper Eckhaus Size M, TRIXIE Heimtierbedarf GmbH, Tarp, Germany; 1× JR Farm Heuhaus 85 g, JR FARM GmbH, Holzheim-Pessenburgheim, Germany). During social and physical environmental enrichment, rats further received two servings of six pieces of sweetened puffed wheat cereal (Kellogg’s Smacks; Kellogg Deutschland, Hamburg, Germany), which were hidden within the cage.

In standard housing and social and physical environmental enrichment conditions, two siblings were always housed with two further pairs of *Cacna1c*^+/–^ and *Cacna1c*^+/+^ siblings from different litters. To avoid age differences between litters, all rats included in the experiment were born within a 4-day time window.

In total, 54 rats were included in the experiment, with *n* = 9 per genotype and experimental housing condition (*Cacna1c*^+/+^-Iso, *Cacna1c*^+/+^-Stand, *Cacna1c*^+/+^-Enr, *Cacna1c*^ + ⁣/−^-Iso, *Cacna1c*^ + ⁣/−^-Stand, *Cacna1c*^+/–^-Enr; short: +/+ Iso, +/+ Stand, +/+ Enr, + ⁣/⁣− Iso, + ⁣/⁣− Stand, + ⁣/⁣− Enr). One *Cacna1c*^+/–^ rat housed under social and physical environmental enrichment conditions died before the end of the experiment and was excluded from data analysis. PFC and HC of the right brain hemispheres were removed on three consecutive days immediately following the 4 weeks of exposure to the experimental housing conditions at ∼2 months of age. All subsequent analyses were conducted blinded to genotype and environmental background.

### Supplementary Genotype Studies

Besides the GxE study, we performed two additional studies. In the first supplementary study, we tested whether brain mitochondrial bioenergetics differs in *Cacna1c*^+/–^ rats and *Cacna1c*^+/+^ littermate controls depending on brain hemisphere. In this study, six male *Cacna1c*^+/–^ rats and six male *Cacna1c*^+/+^ littermate controls exposed to standard housing conditions were included. Brains were removed at ∼2 months of age.

In the second supplementary study, we tested whether brain mitochondrial bioenergetics differs in *Cacna1c*^+/–^ rats and *Cacna1c*^+/+^ littermate controls depending on age and sex. In this study, eight male *Cacna1c*^+/–^ rats and eight male *Cacna1c*^+/+^ littermate controls as well as eight female *Cacna1c*^+/–^ rats and eight female *Cacna1c*^+/+^ littermate controls exposed to standard housing conditions were included. Brains were removed at ∼10 months of age (Figure 1).

Rats included in the supplementary studies were used before weaning to study isolation-induced ultrasonic vocalizations, developmental milestones, and somatosensory reflexes [(Wöhr et al., 2013); not shown].

### Brain Extraction

For brain removal, rats were first deeply anesthetized with isoflurane (Baxter, Unterschleißheim, Germany), and then decapitated and their brains were quickly extracted. The HC was identified and removed as described previously (Sungur et al., 2017), following the gross anatomical criteria established before (Rosen et al., 2000). To expose the dorsal part of the HC, the cerebral cortex covering it was incised and pulled up. The HC was then separated from cortex and removed toward the ventral side. Next, using a cold stainless steel adult rat brain matrix (Zivic Instruments, Pittsburgh, PA, United States), the brain was divided into left and right hemispheres and subsequently the prefrontal cortex (PFC) was isolated via a coronal cut approximately between +2.5 and +5.0 mm from bregma, using the rat brain atlas as anatomical reference (Paxinos and Watson, 2007).

### Mitochondrial Isolation

Mitochondria were isolated from both PFC and HC of acutely dissected adult rat brains using a pump-controlled cell homogenizer (Isobiotec, Heidelberg, Germany) with a clearance of 10 μm and a constant pump flow rate of 700 μl/min as published previously (Schmitt et al., 2013). In brief, fresh brain tissue (∼50 mg), kept in isolation buffer containing 300 mM sucrose, 5 mM TES, 200 μM EGTA, and 1 mM DTT, was coarsely sheared with a 20G Neoject needle (Dispomed, Gelnhausen, Germany) and then strained through a 100 μm nylon cell strainer (Corning Incorporated, Corning, NY, United States). The resulting cell suspension was filled in a 1 ml gas tight glass syringe (Supelco, Munich, Germany) and homogenized with three strokes through the cell homogenizer. Subsequently, the homogenate was centrifuged at 800 × *g* for 10 min at 4°C. The supernatant, referred to as total lysate, was transferred to a new tube and centrifuged at 9,000 × *g*, again for 10 min at 4°C (Heraeus fresco 17, Thermo Fisher Scientific, Darmstadt, Germany). The resulting supernatant contains the cytosolic fraction and the emerging pellet represents the crude mitochondrial fraction which was slowly resuspended in 500 μl mitochondrial isolation buffer (70 mM sucrose, 210 mM mannitol, 5 mM HEPES, 1 mM EGTA, 0.5% (w/v) BSA; pH 7.2). All steps were performed on ice. The total protein concentration was determined using the Pierce BCA Protein Assay Kit (Thermo Fisher Scientific, Darmstadt, Germany) with BSA standards based on mitochondrial isolation buffer.

Unless otherwise stated, all reagents were purchased from Sigma-Aldrich (Munich, Germany). As isolated mitochondria decline in their functional quality over time, our study was strictly littermate controlled and the isolation process was conducted in a randomized order to compensate for potential inter-day and time-dependent variability.

### Protein Analysis

Protein was extracted from frozen prefrontal and hippocampal tissue (10 mg) lysed in buffer containing 50 mM Tris–HCl, 150 mM NaCl, 5 mM EDTA, 1.0% (w/v) Triton X-100, and 0.5% (w/v) Na-deoxycholate, freshly supplemented with 2 mM PMSF, 1 mM Na_3_VO_4_, and 10 mM NaF. Tissue lysis was succeeded by a homogenization step using a T10 basic Ultra-Turrax (IKA-Werke, Staufen, Germany) for 10 s. The homogenates were then centrifuged for 15 min at 13,000 × *g* and 4°C resulting in the protein-comprising supernatants (Heraeus Fresco 17; Thermo Fisher Scientific, Darmstadt, Germany). In addition, the collected fractions from the isolation procedure, i.e., total lysate, cytosolic and mitochondrial fraction, were also used for protein quantitation. The total protein amount was determined with the Pierce BCA Protein Assay Kit (Thermo Fisher Scientific, Darmstadt, Germany). A total of 50 μg protein per sample were loaded on 7.5 and 12.5% polyacrylamide gels, respectively. After electrophoresis, the proteins were transferred onto PVDF membranes (Roche Diagnostics, Mannheim, Germany) and incubated with the respective primary antibodies overnight at 4°C. The following antibodies were used: Ca_*V*_1.2 (1:1,000; Alomone Labs, Jerusalem, Israel) and Vinculin (1:20,000; Sigma-Aldrich, Munich, Germany). Protein detection was achieved using peroxidase labeled secondary antibodies (Vector Laboratories, Burlingame, CA, United States) and luminol based HRP-Juice Plus (PJK GmbH, Kleinblittersdorf, Germany). The resulting chemiluminescence was imaged with a ChemiDoc XRS system (Bio-Rad Laboratories, Hercules, CA, United States). Protein quantification was performed using Bio-Rad Image Lab^TM^ Software.

### Mitochondrial Bioenergetics Measurement

The oxygen consumption rate (OCR) as an indicator of mitochondrial respiration was measured with a Seahorse XF^*e*^96 Analyzer (Agilent Technologies, Waldbronn, Germany). The coupling assay was performed according to existing protocols (Rogers et al., 2011). Briefly, 6 μg of freshly isolated mitochondrial protein per well were assayed in mitochondrial assay solution containing 70 mM sucrose, 220 mM mannitol, 10 mM KH_2_PO_4_, 5 mM MgCl_2_, 2 mM HEPES, 1 mM EGTA, and 0.2% (w/v) fatty-acid-free BSA, with an adjusted pH of 7.2 and supplemented with the complex II substrate succinate (10 mM) and the complex I inhibitor rotenone (2 μM). The consistent adherence of the mitochondria to the well ground was ensured by centrifugation of the whole plate at 2,000 × *g* for 20 min at 4°C (Heraeus Megafuge 40R; Thermo Fisher Scientific, Darmstadt, Germany). The Seahorse system allows for the consecutive injection of different modulators of the electron transport chain. The compounds and final concentrations used are as follows: 4 mM ADP, 2.5 μg/ml oligomycin, 4 μM FCCP, 4 μM antimycin A. Data analysis and parameter calculation were conducted as described previously (Salabei et al., 2014) and in Table 1.

**TABLE 1:** Seahorse parameter calculations.

**Parameter**	**Rate measurement equation**
Anti A	Minimum rate measurement after antimycin A injection
Basal	Last rate measurement before first injection - Anti A
State 3	Maximum rate measurement after ADP injection - Anti A
State 4_*o*_	Minimum rate measurement after oligomycin injection - Anti A
State 3_*u*_	Maximum rate measurement after FCCP injection - Anti A
RCR	Respiratory control ratio = State 3/State 4_*o*_

### Flow Cytometry

Freshly isolated mitochondrial protein was suspended in mitochondrial assay solution at a concentration of 50 μg/ml. The probes were stained with fluorescent dye and incubated on ice for 15 min protected from light. Mitochondrial superoxides were detected by incubation with 1.25 μM MitoSOX Red indicator (Thermo Fisher Scientific, Darmstadt, Germany). Changes in mitochondrial membrane depolarization were determined using MitoPT TMRE Kit (0.2 μM; ImmunoChemistry Technologies, Hamburg, Germany) and with 10 mM succinate and 2 μM rotenone added to the buffer. The samples were measured with a total count of 50,000 events utilizing a Guava easyCyte 6-2L flow cytometer (Merck Millipore, Darmstadt, Germany). Analysis and gating were performed with the GuavaSoft 3.1.1 software.

### Statistical Analysis

Data is presented as single values together with their mean and standard deviation (SD). Analysis of the cortex and the hippocampus were performed separately. The outcome variables studied in the statistical analysis were Ca_*V*_1.2 protein levels, body weight, tissue weight, the parameters of the mitochondrial bioenergetics measurements (Basal, State 3, State 4_*o*_, State 3_*u*_, Anti A, and RCR), ETC complex protein levels (CI-CV), MitoSOX, and TMRE fluorescence. Besides genotype (*Cacna1c*^+/+^/*Cacna1c*^+/–^), explanatory variables included in the analysis were environment (Iso, Stand, Enr), brain hemisphere (left/right), age (2/10 months), and sex (male/female). Each of these factors was fully crossed with the genotype, but none of them were crossed with any of the other explanatory variables. Hence, a separate analysis of each of these variables on the respective outcome variables was performed.

In the case of Ca_*V*_1.2 protein levels, the experimental design (i.e., 3 housing conditions × 2 genotype conditions) was evaluated by a 2-factorial analysis of variance (ANOVA), including the main effects genotype, environment, and the gene-environment interaction. For all other outcome variables, the littermate pairs [one rat with genotype *Cacna1c*^+/+^ (WT), the other with genotype *Cacna1c*^+/–^ (HET)] which were raised under the same environmental (housing) conditions were considered in the analysis. Rather than analyzing the 2-factor cross-over design as a 2-factorial ANOVA

(1) y=i⁢j⁢ka+bxi+1kcxj+2k(bc)xi⁢jx1k+2kεi⁢j⁢k

for rat k with genotype i and housing j, we evaluated the model

(2) y-W⁢T⁢j⁢ky=H⁢E⁢T⁢j⁢ka+bxj+kεj⁢k

for the siblings k (with different genotypes WT and HET) in housing j, instead. In this model, testing for b_*j*_ ≠ 0 is a test for the existence of a gene-environment interaction. A test on a ≠ 0 is a test for the existence of a main genotype effect. The first test in each 2-factorial ANOVA was always a test on the gene-environment interaction. If the hypothesis of no interaction could be rejected, we report results for each genotype x environment combination separately. If neither a significant gene-environment interaction, nor a genotype main effect was present, the environmental main effect was analyzed without consideration of littermate pairs. Residual distributions were visually checked for symmetry and variance homogeneity. All residual distributions had symmetric shape, and only in the data sets of the MitoSOX and TMRE measurements variance heteroscedasticity was present. Generally, the 2-factorial ANOVAs were evaluated by ordinary least squares (OLS) and *F*-tests with the exception of the MitoSOX and TMRE data, in which the 2-factorial ANOVA was evaluated by weighed least squares (WLS) with weights set to the reciprocal of the OLS-residual variance.

All *p*-values are reported up to four digits of significance and were considered statistically significant at *p* < 0.01. The analysis was carried out using R (Version 3.5.2). All statistical models and full result tables are presented in the Supplementary Material. Figures were created with GraphPad Prism (Version 7; GraphPad Software, La Jolla, CA, United States).

## Results

**FIGURE 2:**
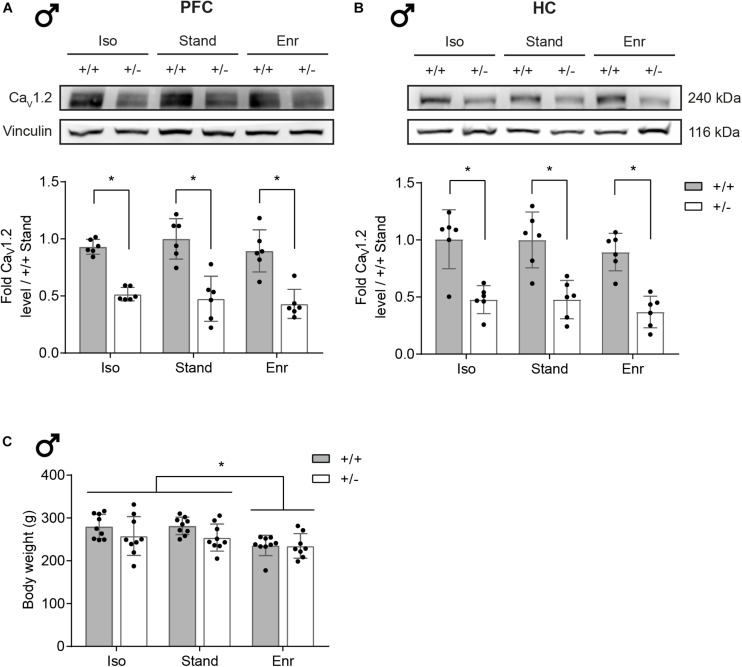
Ca_*V*_1.2 protein levels in rat brain and body weight. Ca_*V*_1.2 protein expression levels in the right prefrontal cortex and hippocampus of 2-month-old male *Cacna1c*^+/–^ rats and *Cacna1c*^+/+^ littermate controls from the different environmental conditions were analyzed by Western blot. **(A)** One representative immunoblot per brain area is shown. **(B)** The bar graphs were obtained by densitometric quantification of the Western blot data. The values are normalized to the loading control vinculin and presented as fold of *Cacna1c*^+/+^-Stand (mean ± SD, *n* = 6). **(C)** Whole body weight was determined after the 4-week exposure to the experimental housing conditions at ∼2 months of age (mean ± SD, *n* = 8–9). Statistical significance is highlighted by an asterisk (^∗^). +/+, wildtype *Cacna1c*^+/+^; + ⁣/⁣−, heterozygous *Cacna1c*^+/–^; Iso, isolation; Stand, standard housing; Enr, enrichment.

In order to study the combined effects of a heterozygous *Cacna1c* knockout and different environmental influences on brain mitochondria, we first validated the reduction of Ca_*V*_1.2 levels in *Cacna1c*^+/–^ rats, compared the mean body weight per group, examined the PFC/HC tissue weight, and verified the functional integrity of the isolated mitochondria. As evident from Figure 2, the Ca_*V*_1.2 protein content is decreased by around 50% in the PFC of *Cacna1c*^+/–^ rats (mean 0.47; 95% confidence interval (CI) 0.41,0.54) compared to their *Cacna1c*^+/+^ littermates (mean 0.94; 95% CI 0.87,1.01), irrespective of the environmental condition (see Supplementary Material for full tables of statistical results). The same applies to the Ca_*V*_1.2 protein levels in the HC of *Cacna1c*^+/–^ (mean 0.44; 95% CI 0.35,0.53) versus *Cacna1c*^+/+^ rats (mean 0.97; 95% CI 0.88,1.06). Statistical analysis found no evidence for a gene-environment interaction (GxE; PFC, *p* = 0.6632; HC, *p* = 0.9989) or for an environmental effect (E; PFC, *p* = 0.4049; HC, *p* = 0.2590), but revealed a significant genetic effect (G; PFC, *p* < 0.0001; HC, *p* < 0.0001). Besides the 240 kDa full-length form of Ca_*V*_1.2, a C-terminally truncated shorter version of the α_1__*C*_-subunit was also affected by the heterozygous *Cacna1c* knockout as shown in Supplementary Figure S1a. Together, these constitute the primary and major size forms of α_1__*C*_ in the brain (Buonarati et al., 2017).

In turn, the housing condition had an impact on the body weight of the 2-month-old rats. Compared to social isolation and standard housing, social and physical environmental enrichment led to a significant weight loss of 33.1 g (95% CI 28.4,37.9) in both *Cacna1c*^+/–^ rats and their *Cacna1c*^+/+^ littermates (E, *p* = 0.0035; G, *p* = 0.0189; GxE, *p* = 0.6312) (Figure 2C). Immediately after its extraction, we also determined the weight of the two brain regions studied (Supplementary Figure S1b). However, since we found no significant genotype- and/or environment-dependent differences in PFC (GxE, *p* = 0.3293; G, *p* = 0.4180; E, *p* = 0.9694; mean 62.7; 95% CI 59.9,65.6) and HC (GxE, *p* = 0.3070; G, *p* = 0.3630; E, *p* = 0.7412; mean 42.0; 95% CI 40.0,43.9) tissue weight, it was not considered in further analyses of mitochondrial function.

The previously established isolation of mitochondria from fresh brain tissue using a speed- and pressure-controlled cell rupture system including a Balch-style homogenizer with a defined clearance constitutes a fast, efficient, and replicable method (Schmitt et al., 2013), which ensured the separation of a crude fraction of functional active mitochondria from nuclear and cytosolic parts (Supplementary Figure S2).

### Mitochondrial Bioenergetics and Respiratory Chain Complex Levels

**FIGURE 3:**
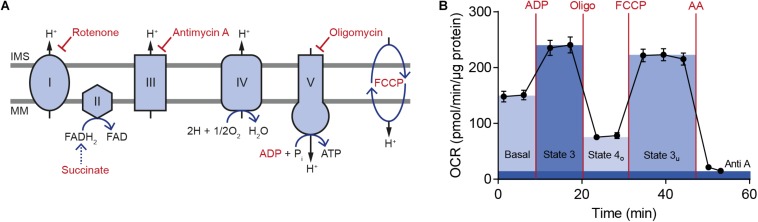
Key steps in oxidative phosphorylation and representative oxygen consumption rate measurement with isolated mitochondria from rat brain. **(A)** The scheme illustrates the electron transport chain with its five different complexes. Modulating compounds used in the Seahorse mitochondrial coupling assay are highlighted in red. **(B)** An exemplary measurement of the oxygen consumption rate (OCR) from isolated rat brain mitochondria is shown together with key parameters of mitochondrial respiration, which were calculated from the data. Five technical replicates are displayed as mean ± SD. IMS, intermembrane space; MM, mitochondrial membrane; OCR, oxygen consumption rate; Oligo, oligomycin; AA, antimycin A.

To compare brain bioenergetics in the GxE study using extracellular flux analysis, mitochondria were isolated from PFC and HC of *Cacna1c*^+/–^ rats and their *Cacna1c*^+/+^ littermates, which were each exposed to one of the three experimental housing conditions, i.e., post-weaning social isolation, standard housing, or social and physical environmental enrichment during the critical developmental period of adolescence (Figure 1). As cellular regulation of mitochondrial function was removed during isolation, energy demand and substrate availability can be specifically modulated to identify site-specific changes in mitochondrial activity and to examine the degree of coupling between the electron transport chain (ETC) and the oxidative phosphorylation machinery. In the performed coupling experiments, respiratory states, indicative of mitochondrial function, were measured as absolute oxygen consumption rate (respiration), normalized to mitochondrial protein content (Figure 3).

**FIGURE 4:**
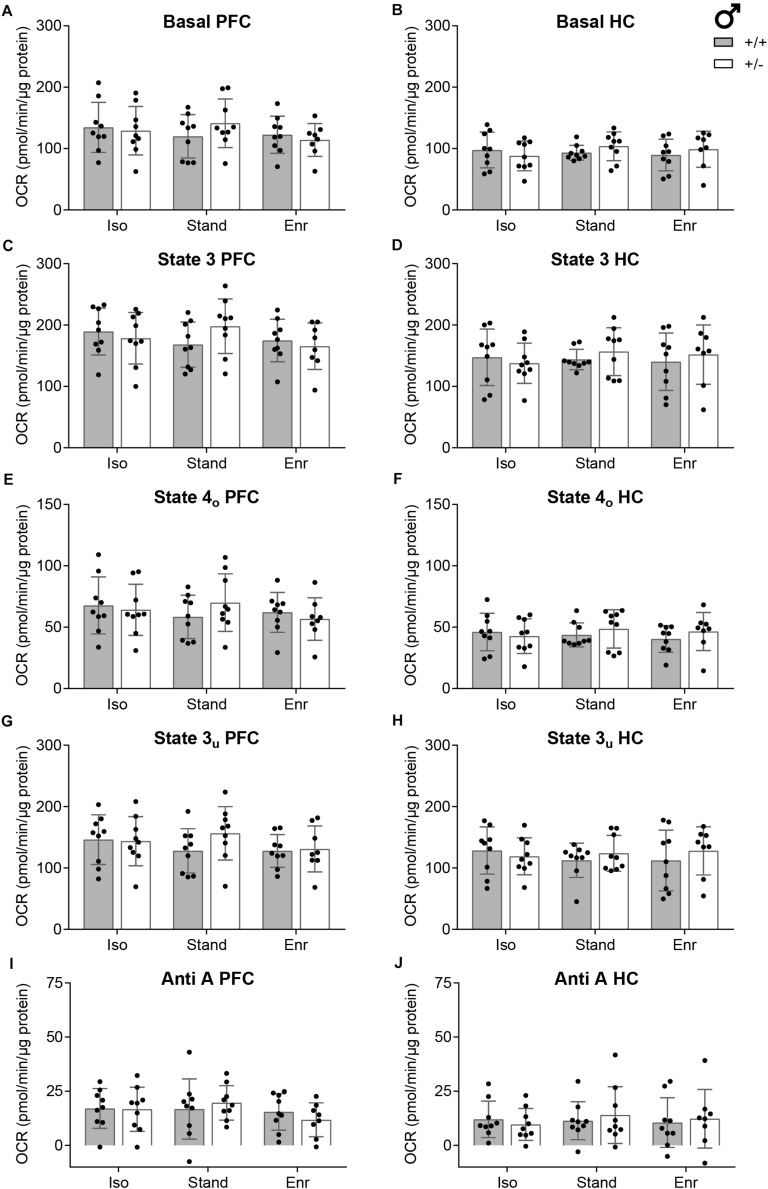
Indices of mitochondrial function calculated from the OCR measurements. Individual parameters of the bioenergetics measurements with isolated mitochondria from rat right prefrontal cortex (PFC; **A,C,E,G,I**) and right hippocampus (HC; **B,D,F,H,J**) are specified. **(A,B)** With rotenone and succinate present in the assay buffer, the Basal rate implies the ADP-independent, complex II-mediated respiration. **(C,D)** The State 3 OCR is initiated with the injection of ADP and accounts for the phosphorylating respiration. **(E,F)** State 4_*o*_ is induced by the addition of the ATP synthase inhibitor oligomycin and stands for the non-phosphorylating respiration. **(G,H)** FCCP uncouples the oxygen consumption from ATP production and is used to assess State 3_*u*_, which represents the maximal respiratory activity. **(I,J)** The Anti A rate constitutes the OCR response to antimycin A which inhibits the mitochondrial respiration. The parameter values are calculated as absolute oxygen consumption rate in pmolO_2_/min/6 μg mitochondrial protein. The data of *n* = 8–9 2-month-old male rats per group are presented as mean ± SD. OCR, oxygen consumption rate; + ⁣/⁣ +, wildtype *Cacna1c*^+/+^ (gray bars); + ⁣/⁣−, heterozygous *Cacna1c*^+/–^ (clear bars); Iso, isolation; Stand, standard housing; Enr, enrichment.

To assess the Basal respiration state, mitochondria were initially incubated with the complex II substrate succinate and, to prevent reverse electron flow, with the complex I inhibitor rotenone (Figures 4A,B). Then, ADP was added to induce phosphorylation and ATP synthesis (State 3; Figures 4C,D). Both parameters did not differ significantly among the six groups, neither in the PFC (Basal; GxE, *p* = 0.0235; G, *p* = 0.5950; E, *p* = 0.4888; State 3; GxE, *p* = 0.0236; G, *p* = 0.6760; E, *p* = 0.5307) nor in the HC (Basal; GxE, *p* = 0.2917; G, *p* = 0.6090; E, *p* = 0.7683; State 3; GxE, *p* = 0.5156; G, *p* = 0.6880; E, *p* = 0.8343), thereby excluding an effect on ATP turnover. Next, State 4_*o*_ was initiated by the injection of oligomycin to inhibit the ATP synthase (complex V). This state allows to assess proton leak and was not affected by either the genotype (G; PFC, *p* = 0.6430; HC, *p* = 0.3560), the environment (E; PFC, *p* = 0.6178; HC, *p* = 0.8159) or their interaction (GxE; PFC, *p* = 0.0332; HC, *p* = 0.3022) in both brain regions studied (Figures 4E,F). After oligomycin addition the protonophore FCCP was injected to dissipate the proton gradient leading to enhanced oxygen consumption, which is not coupled to phosphorylation. Thus, the achieved State 3_*u*_ is exclusively controlled by substrate oxidation. Here, the genotype (G; PFC, *p* = 0.2270; HC, *p* = 0.6070), the environment (E; PFC, *p* = 0.4331; HC, *p* = 0.8917), and their interaction (GxE; PFC, *p* = 0.2116; HC, *p* = 0.5315) also showed no effect on State 3_*u*_ respiration of mitochondria isolated from PFC or HC (Figures 4G,H). The final injection of the complex III inhibitor antimycin A was used to correct all parameters for non-mitochondrial respiration, which was consistently low in all conditions as cytosolic oxidases were eliminated during the isolation process (GxE; PFC, *p* = 0.1301; HC, *p* = 0.3117; G; PFC, *p* = 0.8220; HC, *p* = 0.7310; E; PFC, *p* = 0.3851; HC, *p* = 0.8580) (Figures 4I,J).

**FIGURE 5:**
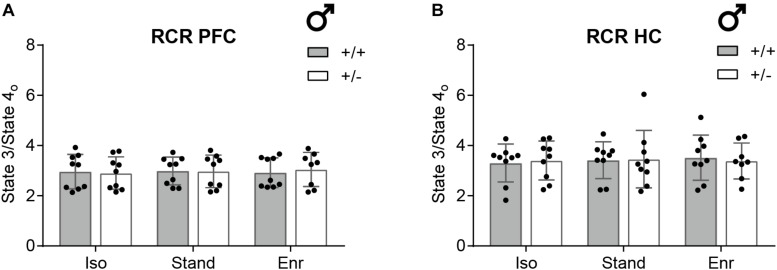
Respiratory control ratio (RCR) as indicator for mitochondrial integrity. The RCR was determined from isolated mitochondria of **(A)** the right prefrontal cortex and **(B)** the right hippocampus of 2-month-old male *Cacna1c*^+/–^ rats and *Cacna1c*^+/+^ littermate controls which were each kept in one of the three different environmental conditions. The State 3 to State 4_*o*_ ratio (RCR) is a measure of the coupling quality between respiration and phosphorylation (mean ± SD, *n* = 8–9). PFC, prefrontal cortex; HC, hippocampus; + / +, wildtype *Cacna1c*^+/+^ (gray bars); + ⁣/⁣−, heterozygous *Cacna1c*^+/–^ (clear bars); Iso, isolation; Stand, standard housing; Enr, enrichment.

In line with previous studies investigating brain region-specific differences in mitochondrial bioenergetics (Pandya et al., 2016), all respiration rates in the HC were lower compared to the PFC with no effect on the respiratory control ratio (Figures 4, 5). The respiratory control ratio (RCR) is defined as the oxygen consumption in State 3 divided by that in State 4_*o*_. This internally normalized parameter provides combined information on substrate oxidation capacity, ATP turnover, and proton leakage making it a potent overall marker of energetic dysfunction (Brand and Nicholls, 2011). In mitochondria from the right PFC (mean RCR 2.97; 95% CI 2.80,3.13) as well as from the right HC (mean RCR 3.42; 95% CI 3.19,3.64) of 2-month-old male rats, we obtained constant RCR values for all the six groups compared (Figure 5). There was no GxE interaction (PFC, *p* = 0.7004; HC, *p* = 0.4914), no genetic (PFC, *p* = 0.8960; HC, *p* = 0.7890), and no environmental effect (PFC, *p* = 0.9640; HC, *p* = 0.9275) on RCR in both, PFC and HC.

To control for potential brain hemisphere-, age-, or sex-related effects, mitochondrial RCRs were also evaluated in right versus left hemisphere, in 2- versus 10-month-old animals, and in female versus male, using *Cacna1c*^+/–^ rats and *Cacna1c*^+/+^ littermates exposed to standard housing conditions in two supplementary studies (Supplementary Figure S3). Our measurements revealed neither a gene-hemisphere interaction (*p* = 0.2740) nor an effect of the hemisphere (*p* = 0.2010) on 2-month-old male PFC RCR alone (Supplementary Figure S3a). Interestingly, in the PFC of 10-month-old normally housed male *Cacna1c*^+/–^ rats, the RCR was moderately, but significantly elevated by 0.45 (95% CI 0.28,0.62) compared to the 3.90 average RCR (95% CI 3.67,4.13) of *Cacna1c*^+/+^ littermate controls (GxA, *p* < 0.0001; G, *p* = 0.0004) (Supplementary Figure S3b). However, no genotype effect on RCR of 10-month-old animals was observed in male HC (G, *p* = 0.3360; mean 4.50; 95% CI 4.11,4.90), female PFC (G, *p* = 0.4260; mean 4.32; 95% CI 3.94,4.69), and female HC (G, *p* = 0.0909; mean 4.35; 95% CI 4.21,4.49), respectively (Supplementary Figures S3c–e). Further, male HC RCRs were significantly higher in the 10- compared with the 2-month-old animals (Age, *p* = 0.0003; GxA, *p* = 0.6583) (Supplementary Figure S3c). No statistically significant differences between sexes were detected; neither in PFC RCR (GxS, *p* = 0.0613; S, *p* = 0.3369) nor in HC RCR (GxS, *p* = 0.0748; S, *p* = 0.2708) (Supplementary Figures S3d,e).

In addition to the functional assessment, we continued to quantify the expression levels of the five ETC complexes by Western blot. Consistently, these showed no changes in relation to genotype and/or environment, neither in the PFC (CI; GxE, *p* = 0.8250; G, *p* = 0.6930; E, *p* = 0.1221; CII; GxE, *p* = 0.3438; G, *p* = 0.4050; E, *p* = 0.8441; CIII; GxE, *p* = 0.5105; G, *p* = 0.1390; E, *p* = 0.5992; CIV; GxE, *p* = 0.9364; G, *p* = 0.7110; E, *p* = 0.7078; CV; GxE, *p* = 0.1485; G, *p* = 0.9990; E, *p* = 0.6015) nor in the HC (CI; GxE, *p* = 0.6014; G, *p* = 0.5580; E, *p* = 0.4120; CII; GxE, *p* = 0.1782; G, *p* = 0.7990; E, *p* = 0.4237; CIII; GxE, *p* = 0.3912; G, *p* = 0.5160; E, *p* = 0.7028; CIV; GxE, *p* = 0.2748; G, *p* = 0.8500; E, *p* = 0.6958; CV; GxE, *p* = 0.6685; G, *p* = 0.3960; E, *p* = 0.4066) (Supplementary Figures S4, S5).

### Mitochondrial Superoxide Levels and Membrane Potential

**FIGURE 6:**
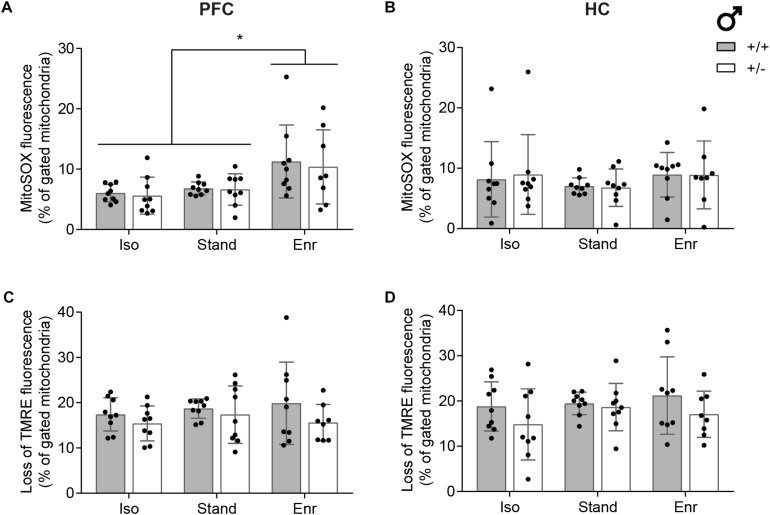
Assessment of mitochondrial superoxide levels and membrane potential. Flow cytometric measurements were conducted with isolated mitochondria from right prefrontal cortex (PFC; **A,C**) and right hippocampus (HC; **B,D**) of 2-month-old male rats using **(A,B)** the mitochondrial superoxide indicator dye MitoSOX and **(C,D)** the mitochondrial membrane potential sensitive dye TMRE. The data was gated to the reference condition *Cacna1c*^+/+^-Stand in each group of six and is displayed as mean ± SD (*n* = 8–9). Statistical significance is highlighted by an asterisk (^∗^). +/+, wildtype *Cacna1c*^+/+^ (gray bars); + ⁣/⁣−, heterozygous *Cacna1c*^+/–^ (clear bars); Iso, isolation; Stand, standard housing; Enr, enrichment.

To further substantiate the results from the bioenergetics analyses, we measured associated mitochondrial parameters including ROS levels and membrane potential (ΔΨ_*m*_) by using flow cytometry (Cottet-Rousselle et al., 2011). The fluorescent dye MitoSOX is rapidly targeted to mitochondria where it is selectively oxidized by superoxide (O_2_^∙−^) producing red fluorescence (Supplementary Figure S6a). O_2_^∙−^ is increasingly formed upon perturbations in the electron flow across the respiratory chain. Moreover, superoxide generation is strongly dependent on the mitochondrial membrane potential, which, in turn, is particularly important for the regulation of ATP synthesis, indicating that all these processes are closely interconnected. The slow-responding cationic dye TMRE (tetramethylrhodamine ethyl ester) accumulates within the mitochondria in a membrane potential-dependent manner and is thereby well suited for measuring pre-existing ΔΨ_*m*_ (Supplementary Figure S6b). As detailed in Figure 6 and consistent with our previous findings, both superoxide levels and ΔΨ_*m*_ of mitochondria isolated from Prefrontal cortex and HC remained unaltered in response to *Cacna1c* haploinsufficiency and different environmental situations with the exception of physical and social enrichment. Here, we found significantly higher mitochondrial ROS levels (mean 10.9; 95% CI 8.0,13.7) versus isolation and standard housing (mean 6.3; 95% CI 5.5,7.0), but only in mitochondria isolated from the PFC and not from the HC. While showing no gene-environment interaction (*p* = 0.7700) and no genetic effect (*p* = 0.3180), mitochondrial ROS levels were significantly higher in the PFC of enriched animals (E, *p* = 0.0062). No statistical significant differences were observed in hippocampal mitochondrial superoxide levels (mean 7.5; 95% CI 6.5,8.5; GxE, *p* = 0.9490; G, *p* = 0.9050; E, *p* = 0.2559) as well as in the membrane potential of mitochondria isolated from PFC (mean 17.2; 95% CI 15.8,18.5; GxE, *p* = 0.6998; G, *p* = 0.0696; E, *p* = 0.5434) and HC (mean 18.6; 95% CI 16.9,20.3; GxE, *p* = 0.4958; G, *p* = 0.0471; E, *p* = 0.5026).

In summary, genotype and environment alone or in interaction did not have a substantial effect on overall mitochondrial bioenergetics in brain regions that are affected in psychiatric disorders (PFC, HC). Furthermore, the functional profile of the isolated brain mitochondria was completed by the evaluation of respiratory chain complex levels, superoxide formation, and membrane potential also not providing evidence for mitochondrial impairment associated with the investigated conditions.

## Discussion

In contrast to our initial evidence-based hypothesis, we could not detect any major differences in brain mitochondrial performance between constitutive haploinsufficient *Cacna1c*^+/–^ rats and wildtype littermates irrespective of the environmental condition, i.e., post-weaning social isolation, standard housing, and social and physical environmental enrichment. By studying different aspects of mitochondrial function such as respiration, ROS production, ΔΨ_*m*_, and ETC-complex protein levels, we repeatedly confirmed that there is no genotype effect, no distinct environment effect, and also no GxE interaction, emphasizing the robustness and reliability of our (negative) results. To our knowledge, a potential link between altered expression of the psychiatric risk gene *Cacna1c* and cerebral mitochondrial function has not been investigated *in vivo* so far. It is well known, however, that the respiratory chain is regulated by the cytosolic calcium concentration (Pizzo et al., 2012), which in turn is very likely dependent on the level of Ca_*V*_1.2 at the cell membrane. Despite a ∼50% reduction in brain Ca_*V*_1.2 levels in the present rat model, the heterozygous *Cacna1c* genotype consistently had no effect on PFC and HC bioenergetics compared to wildtype littermates, independent of the hemisphere and environmental condition studied. One possible reason might be that developmental *Cacna1c* heterozygosis either is not sufficient to perceive detectable changes, or induces adaptive mechanisms facilitating alternative calcium influx routes. In this regard, it has been reported previously that chronic genetic loss of Ca_*V*_1.2 in the central nervous system of mice leads to a compensatory upregulation of calcium-permeable AMPA receptors, thereby maintaining appropriate intracellular calcium concentrations and normal neuronal plasticity (Langwieser et al., 2010).

In the context of GxE interactions, we have shown recently that *Cacna1c* downregulation mediates cellular resilience against oxidative stress in neuronal HT22 cells particularly at the level of mitochondria (Michels et al., 2018a). Furthermore, using different *Cacna1c* siRNAs, our previous findings indicate that a sufficiently strong knockdown of at least 62% in Ca_*V*_1.2 expression is essential to achieve significant protective effects in mouse hippocampal HT22 cells exposed to oxidative stress, including the maintenance of ATP production, respiration, and ΔΨ_*m*_ as well as the prevention of excessive ROS formation (Michels et al., 2018a, b). Consistently, several animal studies have confirmed that modified *Cacna1c* expression modulates stress susceptibility in adult male mice (Terrillion et al., 2017; Dedic et al., 2018). However, in these previous studies, mitochondrial parameters were not analyzed. For example, the region-specific deletion of *Cacna1c* in the nucleus accumbens was associated with increased stress sensitivity in mice exposed to chronic social defeat for 10 days (Terrillion et al., 2017). Accordingly, *Cacna1c* depletion in forebrain glutamatergic neurons during embryonic development promoted susceptibility to chronic stress in mice subjected to social defeat for 3 weeks (Dedic et al., 2018). Conversely, 4 weeks of chronic unpredictable stress induced depressive- and anxiety-like behavior in both *Cacna1c* heterozygous mice and their wildtype littermates when assessed 1–2 days following stress (Bavley et al., 2017). Similar to this unchanged performance in PFC-dependent behavioral tasks, *Cacna1c* haploinsufficiency did not interact with 4-week early life social isolation stress to affect PFC or HC mitochondrial bioenergetics in the present rat study. Discrepancies in the above-mentioned findings might be related to different species, sites of the genetic modification, age, stress paradigms and duration, or readouts. Environmental enrichment was initially intended for rescuing potential adverse genotype effects on mitochondrial performance and had neither a combined gene-environment impact, nor a beneficial influence on its own in the current experiments. However, physical and social environmental enrichment showed a general effect on the rats’ biometric characteristics and ROS generation. In line with prior studies, environmentally enriched animals had significantly lower body weights than their non-enriched controls, possibly attributed to a higher physical activity and most likely influenced by the type and duration of enrichment (Sparling et al., 2018). Moreover, 4 weeks of environmental enrichment resulted in significantly increased mitochondrial ROS levels in the rats’ prefrontal cortex, constituting a common response of tissues to physical exercise (Radak et al., 2016).

Compared to *Cacna1c*-associated effects, the impact of chronic stress alone on brain mitochondrial function has already been examined in a number of rodent studies (for review see Klinedinst and Regenold, 2015; Picard and McEwen, 2018). Traditional chronic stress paradigms, including chronic unpredictable stress (CUS) for 6 weeks and chronic restraint stress (CRS) for 2–3 weeks, are established animal models of depression. Published findings indicate that CUS in adult male rats is accompanied by inhibited activities of the mitochondrial respiratory chain complexes I, III, and IV in whole tissue homogenates from cerebral cortex and cerebellum, but not from PFC and HC (Rezin et al., 2008). Under the same conditions, another study described decreased complex I and IV activities in isolated PFC and HC mitochondria (Liu and Zhou, 2012). Opposing results, however, showed increased complex I, II, and III activities in PFC tissue of adult Wistar rats exposed to CUS (Ortmann et al., 2016). In mice, CUS led to a reduced respiratory control ratio in isolated mitochondria from cortex and HC (Gong et al., 2011). Using the CRS model, further research revealed decreases in enzyme activity of complex I-III without affecting complex IV activity, oxygen consumption, and ATP production in isolated cortical rat mitochondria (Madrigal et al., 2001). CRS in mice caused complex IV inhibition in mitochondria isolated from HC (Seo et al., 2011) and diminished complex I-driven State 3 respiration in isolated forebrain mitochondria (Kambe and Miyata, 2015). Collectively, variations in stress induction paradigms, brain region selection, tissue processing, method application, and parameter evaluation probably contribute to the diverging and in part conflicting results regarding the impact of chronic stress on mitochondrial outcomes. Furthermore, existing studies largely focused on ETC complex activity measurements and observed in most cases decreased activities with effects ranging between 30 and 70% (Picard and McEwen, 2018). However, it has been demonstrated in isolated non-synaptic rat mitochondria that complex I, III and IV activities could be reduced by more than 60% before respiration and ATP synthesis are considerably affected (Davey and Clark, 1996). This suggests that moderate changes in candidate complex activities have little effect on the overall capability of mitochondria to maintain brain energy homeostasis, which is dependent on the integrity of many processes. On the contrary, the oxygen consumption and RCR, which were measured in our and some previous studies, represent a more comprehensive and revealing option to detect bioenergetic dysfunction in isolated mitochondria (Brand and Nicholls, 2011).

With 4 weeks of post-weaning social isolation, we used a different model of environmental stress and found no changes in mitochondrial respiration, membrane potential, ROS formation, or protein levels of the ETC complexes in rat PFC and HC compared to the socially reared littermate controls. Such chronic social isolation represents a mild and more natural stressor than CUS and CRS, which still evokes a variety of neurobehavioral changes in rodents comparable to those observed in patients with psychiatric disorders, including anxiety and depression (Filipović et al., 2017). As reported previously, 4 weeks of juvenile social isolation stress led to decreased ATP and elevated ROS levels in the HC of male mice (Amiri et al., 2015). Post-weaning social isolation for 8 weeks also resulted in reduced ATP accumulation in the frontal cortex, but not in the striatum of male Sprague Dawley rats (Möller et al., 2013). In addition, male Wistar rats showed slightly increased complex IV activity as well as unchanged mitochondrial membrane potential and ROS production in the PFC after 1-week social isolation during the pre-pubertal period (Krolow et al., 2012). All these parameters, which were used as measures of mitochondrial function, were assessed from whole frozen tissue, whereas our experiments were predominantly conducted with isolated fresh mitochondria. Thus, the published measurements are solely normalized to the total protein content in the tissue homogenates, making it impossible to distinguish if the existent findings are based on altered mitochondrial quantity or quality. Whilst the meaningful normalization to mitochondrial protein allowed us to specifically evaluate the functional status of brain mitochondria. Besides varying stress duration and assays used, this apparent difference in the normalization procedure might be a further explanation for the inconsistent findings and additionally impedes the comparability of the studies.

Prefrontal cortex and HC are among the most frequently studied brain regions in neuropsychiatric disorders based on their documented stress sensitivity, especially during the juvenile developmental period and their particular implication in disease-relevant neuronal circuits (Carlson et al., 2006). Nonetheless, while providing brain-region-specific analyses of PFC and HC mitochondria, our work did not differentiate between cells or cell subtypes, sharing this limitation also with all relevant prior studies. Mitochondria isolated from brain tissue originate from different cell types including neuronal and glial cells. This heterogeneous composition of cells could mask potential cell type-dependent variations in stress susceptibility and changes in the glia-neuron ratio. Furthermore, the expression of Ca_*V*_1.2 varies between excitable and non-excitable cells (Harraz and Altier, 2014), which might thus be differently affected by the heterozygous *Cacna1c* knockout. The investigation of cell type-specific mitochondrial bioenergetics therefore constitutes a key challenge for future studies.

Although brain mitochondrial function was not affected or adaptive mechanisms were not yet exhausted in our particular risk GxE setting, i.e., *Cacna1c* haploinsufficiency and post-weaning social isolation, we are still convinced that mitochondria represent an important intersection point between genetic alternations, psychosocial experiences and abnormalities in cerebral energy metabolism, synaptic plasticity in psychiatric disorders (Manji et al., 2012). The overall inconsistent findings in this research field highlight the complexity and heterogeneity of those illnesses and suggest that study results are strongly dependent on many factors, such as animal species, experimental design, and analysis methods. Hence, additional investigations are required to determine the disease-relevant effects on bioenergetics also including long-term studies of GxE interactions in both sexes, since we found a significant effect of *Cacna1c* haploinsufficiency on mitochondrial respiration in the PFC of 10-month-old male, but not female rats, which was not evident in the 2-month-old animals. The extent of this observed genotype effect under standard housing conditions might further vary in combination with different environmental situations and may overall contribute to our understanding of the pathophysiology of neuropsychiatric disorders.

## Ethics Statement

This study was carried out in accordance with the recommendations of the National Institutes of Health Guidelines for the Care and Use of Laboratory Animals and the relevant local or national rules and regulations of Germany. The protocol was approved by the local government (MR 20/35 Nr. 19/2014; Tierschutzbehörde, Regierungspräsidium Gießen, Germany).

## Author Contributions

SM, AMD, MW, RS, and CC designed the study. SW and MR performed the genotyping of the rat strain. SM, AMD, MB, TK, and AS conducted the experiments. SM analyzed the data and wrote the manuscript with the help of all authors. AD performed the statistical analyses. AMD, SW, MR, AD, MW, RS, and CC supervised the project and acquired funding. All authors read and approved the final manuscript.

## Conflict of Interest

The authors declare that the research was conducted in the absence of any commercial or financial relationships that could be construed as a potential conflict of interest.
