# Supplementary material for: Interaction of the Psychiatric Risk Gene Cacna1c With Post-weaning Social Isolation or Environmental Enrichment Does Not Affect Brain Mitochondrial Bioenergetics in Rats
Source: Front Cell Neurosci. 2019 Oct 25;13:483. doi: 10.3389/fncel.2019.00483 (PMC6823196; doi:10.3389/fncel.2019.00483)
Supplement: Supplementary file 1 [file Data_Sheet_1.docx]

Supplementary Material

**Supplementary Materials and Methods**

**Protein analysis**

The following antibodies were used: Ca_V_1.2 (1:1,000; Alomone Labs, Jerusalem, Israel), Vinculin (1:20,000; Sigma-Aldrich, Munich, Germany), Lamin A/C (1:1,000; Cell Signaling Technology, Frankfurt, Germany), α-Tubulin (1:10,000), Tim 23 (1:2,000; BD Bioscience, Heidelberg, Germany), Mitofusin 2 (Mfn2; 1:1,000; Cell Signaling Technology, Frankfurt, Germany), and MitoProfile Total OXPHOS Rodent WB Antibody Cocktail (1:250; Abcam, Cambridge, UK). MitoProfile contains a premixed cocktail of antibodies against the complex I subunit NDUFB8, the iron-sulfur protein subunit SDHB of complex II, the core protein 2 UQCRC2 of complex III, the complex IV subunit MTCO1, and the alpha subunit ATP5A of complex V.

**Rhodamine 123**

Mitochondrial membrane potential (ΔΨ_m_) integrity was assessed over time (40 min) by rhodamine 123 (Thermo Fisher Scientific, Darmstadt, Germany) fluorescence quenching, as previously reported (Zamzami N, et al. Meth Enzymol. 2000). Mitochondrial protein samples (75 µg/well) were resuspended in 200 mM sucrose, 10 mM MOPS-Tris, 5 mM succinate, 1 mM P_i_ (H_3_PO_4_), 10 µM EGTA, and 2 µM rotenone (pH 7.3) and dyed with 125 nM rhodamine 123 in a black 96-well plate (Greiner Bio-One GmbH, Frickenhausen, Germany). Injection of the depolarizing reagent FCCP (500 nM) at the end of the measurement served as internal positive control leading to a complete loss of ΔΨ_m_. The resulting fluorescence signals were detected with a FLUOstar OPTIMA reader (Ex 485 BP12, Em 530 BP10; BMG Labtech, Ortenberg, Germany).

## Microscopy

To visualize mitochondria, the enriched fractions resulting from the isolation procedure were incubated with 0.2 µM MitoTracker Deep Red (Cell Signaling Technology, Frankfurt, Germany) for 15 min. Thereafter, images were acquired using a fluorescence microscope with a HCX PL Fluotar L 40.0x/0.60 dry objective (DMI6000 B; Leica Microsystems, Wetzlar; Germany).

**Supplementary Statistical Material**

## Cav1.2 protein levels PFC

#### Experimental Design

| Sex | Age (Months) | Environment | Genotype | Count |
| --- | --- | --- | --- | --- |
| Male | 2 | Iso | +/+ | 6 |
|  |  |  | +/- | 6 |
|  |  | Stand | +/+ | 6 |
|  |  |  | +/- | 6 |
|  |  | Enr | +/+ | 6 |
|  |  |  | +/- | 6 |

#### Test of gene-environment interaction

Analysis of Variance Table

Response: Cav12
 Df Sum Sq Mean Sq F value Pr(>F)
Genotype 1 6.2277 6.2277 92.2599 1.159e-10
Environment 2 0.1210 0.0605 0.8961 0.4188
Genotype:Environment 2 0.0562 0.0281 0.4164 0.6632
Residuals 30 2.0251 0.0675

R-squared = 0.76

#### Test of genotype and environment main effects

Analysis of Variance Table

Response: Cav12
 Df Sum Sq Mean Sq F value Pr(>F)
Genotype 1 6.2277 6.2277 95.7527 3.845e-11
Environment 2 0.1210 0.0605 0.9301 0.4049
Residuals 32 2.0813 0.0650

R-squared = 0.753

#### Test of genotype effect, final model

Analysis of Variance Table

Response: Cav12
 Df Sum Sq Mean Sq F value Pr(>F)
Genotype 1 6.2277 6.2277 96.148 1.924e-11
Residuals 34 2.2022 0.0648

R-squared = 0.739

Estimate Std. Error t value Pr(>|t|)
(Intercept) 0.842 0.0600 14.03 1.05e-15
GenotypeWT 0.832 0.0848 9.81 1.92e-11

#### Group means

Mean CI: [0.025, 0.975]
+/- 0.474 0.41 0.542
+/+ 0.942 0.87 1.011

## Cav1.2 protein levels HC

#### Experimental Design

| Sex | Age (Months) | Environment | Genotype | Count |
| --- | --- | --- | --- | --- |
| Male | 2 | Iso | +/+ | 6 |
|  |  |  | +/- | 6 |
|  |  | Stand | +/+ | 6 |
|  |  |  | +/- | 6 |
|  |  | Enr | +/+ | 6 |
|  |  |  | +/- | 6 |

#### Test of gene-environment interaction

Analysis of Variance Table

Response: Cav12
 Df Sum Sq Mean Sq F value Pr(>F)
Genotype 1 2.48443 2.48443 69.0673 2.82e-09
Environment 2 0.09507 0.04754 1.3215 0.2818
Genotype:Environment 2 0.00008 0.00004 0.0011 0.9989
Residuals 30 1.07913 0.03597

R-squared = 0.705

#### Test of genotype and environment main effects

Analysis of Variance Table

Response: Cav12
 Df Sum Sq Mean Sq F value Pr(>F)
Genotype 1 2.48443 2.48443 73.6665 8.286e-10
Environment 2 0.09507 0.04754 1.4095 0.259
Residuals 32 1.07921 0.03373

R-squared = 0.705

#### Test of genotype effect

Analysis of Variance Table

Response: Cav12
 Df Sum Sq Mean Sq F value Pr(>F)
Genotype 1 2.4844 2.48443 71.934 6.623e-10
Residuals 34 1.1743 0.03454

R-squared = 0.679

Estimate Std. Error t value Pr(>|t|)
(Intercept) 0.441 0.0438 10.07 9.74e-12
GenotypeWT 0.525 0.0619 8.48 6.62e-10

#### Group means

Mean CI: [0.025, 0.975]
+/- 0.441 0.352 0.53
+/+ 0.967 0.878 1.06

## Bodyweight

G x E is evaluated in a paired design (difference between +/+ and +/- littermate pairs), environmental effect in this design is equivalent to G x E.
If no G x E is present, genotype effect is evaluated in the same design (equivalent to a paired t-test), intercept is equivalent to genotype effect.
If no G x E and no genotype effect are present, the environment (housing) effect is evaluated in all animals (n=53), without considering the littermate pairs.
In the final model, isolation and standard housing were combined into one group, as they had almost identical mean body weight.

#### Experimental Design

| Sex | Age (Months) | Environment | Genotype | Count |
| --- | --- | --- | --- | --- |
| Male | 2 | Iso | +/+ - +/- | 9 |
|  |  | Stand | +/+ - +/- | 9 |
|  |  | Enr | +/+ - +/- | 8 |

#### Test of gene-environment interaction

Analysis of Variance Table

Response: Bodyweight
 Df Sum Sq Mean Sq F value Pr(>F)
Environment 2 1583 791.27 0.4694 0.6312
Residuals 23 38771 1685.68

R-squared = 0.0392

#### Test of genotype main effect

Estimate Std. Error t value Pr(>|t|)
(Intercept) -19.8 7.88 -2.51 0.0189

R-squared = 0

#### Experimental Design

| Sex | Age (Months) | Environment | Genotype | Count |
| --- | --- | --- | --- | --- |
| Male | 2 | Iso | +/+ | 9 |
|  |  |  | +/- | 9 |
|  |  | Stand | +/+ | 9 |
|  |  |  | +/- | 9 |
|  |  | Enr | +/+ | 9 |
|  |  |  | +/- | 8 |

#### Test of environment main effect

Analysis of Variance Table

Response: Bodyweight
 Df Sum Sq Mean Sq F value Pr(>F)
Environment 2 12693 6346.6 6.3515 0.003484
Residuals 50 49962 999.2

R-squared = 0.203

#### Final model

Estimate Std. Error t value Pr(>|t|)
(Intercept) 235.3 7.59 31.0 9.46e-35
Env2ISO/STAND 33.1 9.21 3.6 7.26e-04

#### Group means

Mean CI: [0.025, 0.975]
Enriched 235.3 220.0 250.5
Isolation/Standard 268.4 257.9 278.9

## Tissue Weight PFC

G x E is evaluated in a paired design (difference between +/+ and +/- littermate pairs), environmental effect in this design is equivalent to G x E.
If no G x E is present, genotype effect is evaluated in the same design (equivalent to a paired t-test), intercept is equivalent to genotype effect.
If no G x E and no genotype effect are present, the environment (housing) effect is evaluated in all animals (n=53), without considering the littermate pairs.

#### Experimental Design

| Sex | Age (Months) | Environment | Genotype | Count |
| --- | --- | --- | --- | --- |
| Male | 2 | Iso | +/+ - +/- | 9 |
|  |  | Stand | +/+ - +/- | 9 |
|  |  | Enr | +/+ - +/- | 8 |

#### Test of gene-environment interaction

Analysis of Variance Table

Response: TissueWeight
 Df Sum Sq Mean Sq F value Pr(>F)
Environment 2 479.5 239.73 1.1663 0.3293
Residuals 23 4727.7 205.55

R-squared = 0.0921

#### Test of genotype main effect

Estimate Std. Error t value Pr(>|t|)
(Intercept) -2.33 2.83 -0.823 0.418

R-squared = 0

#### Experimental Design

| Sex | Age (Months) | Environment | Genotype | Count |
| --- | --- | --- | --- | --- |
| Male | 2 | Iso | +/+ | 9 |
|  |  |  | +/- | 9 |
|  |  | Stand | +/+ | 9 |
|  |  |  | +/- | 9 |
|  |  | Enr | +/+ | 9 |
|  |  |  | +/- | 8 |

#### Test of environment main effect

Analysis of Variance Table

Response: TissueWeight
 Df Sum Sq Mean Sq F value Pr(>F)
Environment 2 6.9 3.46 0.0311 0.9694
Residuals 50 5559.6 111.19

R-squared = 0.00124

#### Overall mean

Mean CI: [0.025, 0.975]
All 62.7 59.9 65.6

## Tissue Weight HC

G x E is evaluated in a paired design (difference between +/+ and +/- littermate pairs), environmental effect in this design is equivalent to G x E.
If no G x E is present, genotype effect is evaluated in the same design (equivalent to a paired t-test), intercept is equivalent to genotype effect.
If no G x E and no genotype effect are present, the environment (housing) effect is evaluated in all animals (n=53), without considering the littermate pairs.

#### Experimental Design

| Sex | Age (Months) | Environment | Genotype | Count |
| --- | --- | --- | --- | --- |
| Male | 2 | Iso | +/+ - +/- | 9 |
|  |  | Stand | +/+ - +/- | 9 |
|  |  | Enr | +/+ - +/- | 8 |

#### Test of gene-environment interaction

Analysis of Variance Table

Response: TissueWeight
 Df Sum Sq Mean Sq F value Pr(>F)
Environment 2 217.05 108.526 1.2438 0.307
Residuals 23 2006.80 87.252

R-squared = 0.0976

#### Test of genotype main effect

Estimate Std. Error t value Pr(>|t|)
(Intercept) -1.72 1.85 -0.927 0.363

R-squared = 0

#### Experimental Design

| Sex | Age (Months) | Environment | Genotype | Count |
| --- | --- | --- | --- | --- |
| Male | 2 | Iso | +/+ | 9 |
|  |  |  | +/- | 9 |
|  |  | Stand | +/+ | 9 |
|  |  |  | +/- | 9 |
|  |  | Enr | +/+ | 9 |
|  |  |  | +/- | 8 |

#### Test of environment main effect

Analysis of Variance Table

Response: TissueWeight
 Df Sum Sq Mean Sq F value Pr(>F)
Environment 2 32.09 16.043 0.3013 0.7412
Residuals 50 2662.69 53.254

R-squared = 0.0119

#### Overall mean

Mean CI: [0.025, 0.975]
All 42 40 43.9

## R123 rates PFC

#### Experimental Design

| Sex | Age (Months) | Environment | Genotype | Count |
| --- | --- | --- | --- | --- |
| Male | 10 | Stand | +/+ | 8 |
|  |  |  | +/- | 8 |

Difference between time points t0 and t30 within each animal (paired t-test).

Estimate Std. Error t value Pr(>|t|)
(Intercept) 0.0188 0.00746 2.52 0.0235

R-squared = 0

Mean CI: [0.025, 0.975]
All 0.0188 0.00291 0.0347

## R123 rates HC

#### Experimental Design

| Sex | Age (Months) | Environment | Genotype | Count |
| --- | --- | --- | --- | --- |
| Male | 10 | Stand | +/+ | 8 |
|  |  |  | +/- | 8 |

Difference between time points t0 and t30 within each animal (paired t-test).

#### Test of genotype effect

Estimate Std. Error t value Pr(>|t|)
(Intercept) 0.0272 0.00949 2.87 0.0117

R-squared = 0

Mean CI: [0.025, 0.975]
All 0.0272 0.00699 0.0474

## Bioenergetic parameters PFC

G x E is evaluated in a paired design (difference between +/+ and +/- littermate pairs), environmental effect in this design is equivalent to G x E.
If no G x E is present, genotype effect is evaluated in the same design (equivalent to a paired t-test), intercept is equivalent to genotype effect.
If no G x E and no genotype effect are present, the environment (housing) effect is evaluated in all animals (n=53), without considering the littermate pairs.

#### Experimental Design for test of gene-environment interaction and genotype main effect

| Sex | Age (Months) | Environment | Genotype | Count |
| --- | --- | --- | --- | --- |
| Male | 2 | Iso | +/+ - +/- | 9 |
|  |  | Stand | +/+ - +/- | 9 |
|  |  | Enr | +/+ - +/- | 8 |

#### Experimental Design for test of environment main effect

| Sex | Age (Months) | Environment | Genotype | Count |
| --- | --- | --- | --- | --- |
| Male | 2 | Iso | +/+ | 9 |
|  |  |  | +/- | 9 |
|  |  | Stand | +/+ | 9 |
|  |  |  | +/- | 9 |
|  |  | Enr | +/+ | 9 |
|  |  |  | +/- | 8 |

### Basal

#### Test of gene-environment interaction

Analysis of Variance Table

Response: Basal
 Df Sum Sq Mean Sq F value Pr(>F)
Environment 2 4802 2401.0 4.4323 0.02354
Residuals 23 12459 541.7

R-squared = 0.278

#### Test of genotype main effect

Estimate Std. Error t value Pr(>|t|)
(Intercept) 2.78 5.15 0.539 0.595

R-squared = 0

#### Test of environment main effect

Analysis of Variance Table

Response: Basal
 Df Sum Sq Mean Sq F value Pr(>F)
Environment 2 1834 917.11 0.7262 0.4888
Residuals 50 63147 1262.94

R-squared = 0.0282

#### Overall mean

Mean CI: [0.025, 0.975]
All 127.2 117.5 137

### State3

#### Test of gene-environment interaction

Analysis of Variance Table

Response: State3
 Df Sum Sq Mean Sq F value Pr(>F)
Environment 2 9891.2 4945.6 4.4316 0.02355
Residuals 23 25667.9 1116.0

R-squared = 0.278

#### Test of genotype main effect

Estimate Std. Error t value Pr(>|t|)
(Intercept) 3.12 7.4 0.422 0.676

R-squared = 0

#### Test of environment main effect

Analysis of Variance Table

Response: State3
 Df Sum Sq Mean Sq F value Pr(>F)
Environment 2 1975 987.66 0.6416 0.5307
Residuals 50 76971 1539.41

R-squared = 0.025

#### Overall mean

Mean CI: [0.025, 0.975]
All 179.2 168.5 190

### State4o

#### Test of gene-environment interaction

Analysis of Variance Table

Response: State4o
 Df Sum Sq Mean Sq F value Pr(>F)
Environment 2 1448.7 724.37 3.9619 0.03323
Residuals 23 4205.1 182.83

R-squared = 0.256

#### Test of genotype main effect

Estimate Std. Error t value Pr(>|t|)
(Intercept) 1.38 2.95 0.469 0.643

R-squared = 0

#### Test of environment main effect

Analysis of Variance Table

Response: State4o
 Df Sum Sq Mean Sq F value Pr(>F)
Environment 2 382.2 191.11 0.4862 0.6178
Residuals 50 19652.7 393.05

R-squared = 0.0191

#### Overall mean

Mean CI: [0.025, 0.975]
All 63.27 57.86 68.68

### State3u

#### Test of gene-environment interaction

Analysis of Variance Table

Response: State3u
 Df Sum Sq Mean Sq F value Pr(>F)
Environment 2 4985 2492.3 1.6628 0.2116
Residuals 23 34474 1498.8

R-squared = 0.126

#### Test of genotype main effect

Estimate Std. Error t value Pr(>|t|)
(Intercept) 9.66 7.79 1.24 0.227

R-squared = 0

#### Test of environment main effect

Analysis of Variance Table

Response: State3u
 Df Sum Sq Mean Sq F value Pr(>F)
Environment 2 2401 1200.5 0.8511 0.4331
Residuals 50 70531 1410.6

R-squared = 0.0329

#### Overall mean

Mean CI: [0.025, 0.975]
All 138.9 128.6 149.3

### AntiA

#### Test of gene-environment interaction

Analysis of Variance Table

Response: AntiA
 Df Sum Sq Mean Sq F value Pr(>F)
Environment 2 184.79 92.395 2.2311 0.1301
Residuals 23 952.48 41.412

R-squared = 0.162

#### Test of genotype main effect

Estimate Std. Error t value Pr(>|t|)
(Intercept) -0.301 1.32 -0.228 0.822

R-squared = 0

#### Test of environment main effect

Analysis of Variance Table

Response: AntiA
 Df Sum Sq Mean Sq F value Pr(>F)
Environment 2 181.0 90.487 0.9726 0.3851
Residuals 50 4651.7 93.034

R-squared = 0.0374

#### Overall mean

Mean CI: [0.025, 0.975]
All 16.31 13.65 18.97

## Bioenergetic parameters Hippocampus

G x E is evaluated in a paired design (difference between +/+ and +/- littermate pairs), environmental effect in this design is equivalent to G x E.
If no G x E is present, genotype effect is evaluated in the same design (equivalent to a paired t-test), intercept is equivalent to genotype effect.
If no G x E and no genotype effect are present, the environment (housing) effect is evaluated in all animals (n=53), without considering the littermate pairs.

#### Experimental Design for test of gene-environment interaction and genotype main effect

| Sex | Age (Months) | Environment | Genotype | Count |
| --- | --- | --- | --- | --- |
| Male | 2 | Iso | +/+ - +/- | 9 |
|  |  | Stand | +/+ - +/- | 9 |
|  |  | Enr | +/+ - +/- | 8 |

#### Experimental Design for test of environment main effect

| Sex | Age (Months) | Environment | Genotype | Count |
| --- | --- | --- | --- | --- |
| Male | 2 | Iso | +/+ | 9 |
|  |  |  | +/- | 9 |
|  |  | Stand | +/+ | 9 |
|  |  |  | +/- | 9 |
|  |  | Enr | +/+ | 9 |
|  |  |  | +/- | 8 |

### Basal

#### Test of gene-environment interaction

Analysis of Variance Table

Response: Basal
 Df Sum Sq Mean Sq F value Pr(>F)
Environment 2 2170.2 1085.10 1.3005 0.2917
Residuals 23 19190.8 834.38

R-squared = 0.102

#### Test of genotype main effect

Estimate Std. Error t value Pr(>|t|)
(Intercept) 2.97 5.73 0.518 0.609

R-squared = 0

#### Test of environment main effect

Analysis of Variance Table

Response: Basal
 Df Sum Sq Mean Sq F value Pr(>F)
Environment 2 314 156.98 0.265 0.7683
Residuals 50 29621 592.42

R-squared = 0.0105

#### Overall mean

Mean CI: [0.025, 0.975]
All 95.18 88.56 101.8

### State3

#### Test of gene-environment interaction

Analysis of Variance Table

Response: State3
 Df Sum Sq Mean Sq F value Pr(>F)
Environment 2 2536 1267.9 0.6819 0.5156
Residuals 23 42764 1859.3

R-squared = 0.056

#### Test of genotype main effect

Estimate Std. Error t value Pr(>|t|)
(Intercept) 3.39 8.35 0.406 0.688

R-squared = 0

#### Test of environment main effect

Analysis of Variance Table

Response: State3
 Df Sum Sq Mean Sq F value Pr(>F)
Environment 2 550 274.97 0.1818 0.8343
Residuals 50 75614 1512.27

R-squared = 0.00722

#### Overall mean

Mean CI: [0.025, 0.975]
All 146.1 135.6 156.7

### State4o

#### Test of gene-environment interaction

Analysis of Variance Table

Response: State4o
 Df Sum Sq Mean Sq F value Pr(>F)
Environment 2 491.3 245.64 1.2613 0.3022
Residuals 23 4479.2 194.75

R-squared = 0.0988

#### Test of genotype main effect

Estimate Std. Error t value Pr(>|t|)
(Intercept) 2.6 2.77 0.941 0.356

R-squared = 0

#### Test of environment main effect

Analysis of Variance Table

Response: State4o
 Df Sum Sq Mean Sq F value Pr(>F)
Environment 2 74.3 37.157 0.2043 0.8159
Residuals 50 9093.5 181.869

R-squared = 0.00811

#### Overall mean

Mean CI: [0.025, 0.975]
All 44.59 40.93 48.25

### State3u

#### Test of gene-environment interaction

Analysis of Variance Table

Response: State3u
 Df Sum Sq Mean Sq F value Pr(>F)
Environment 2 2713 1356.3 0.6497 0.5315
Residuals 23 48011 2087.4

R-squared = 0.0535

#### Test of genotype main effect

Estimate Std. Error t value Pr(>|t|)
(Intercept) 4.6 8.83 0.521 0.607

R-squared = 0

#### Test of environment main effect

Analysis of Variance Table

Response: State3u
 Df Sum Sq Mean Sq F value Pr(>F)
Environment 2 297 148.34 0.1149 0.8917
Residuals 50 64533 1290.67

R-squared = 0.00458

#### Overall mean

Mean CI: [0.025, 0.975]
All 120.5 110.7 130.2

### AntiA

#### Test of gene-environment interaction

Analysis of Variance Table

Response: AntiA
 Df Sum Sq Mean Sq F value Pr(>F)
Environment 2 114.62 57.309 1.227 0.3117
Residuals 23 1074.24 46.706

R-squared = 0.0964

#### Test of genotype main effect

Estimate Std. Error t value Pr(>|t|)
(Intercept) 0.471 1.35 0.348 0.731

R-squared = 0

#### Test of environment main effect

Analysis of Variance Table

Response: AntiA
 Df Sum Sq Mean Sq F value Pr(>F)
Environment 2 33.1 16.562 0.1536 0.858
Residuals 50 5390.6 107.812

R-squared = 0.00611

#### Overall mean

Mean CI: [0.025, 0.975]
All 11.62 8.807 14.44

## RCR PFC

G x E is evaluated in a paired design (difference between +/+ and +/- littermate pairs), environmental effect in this design is equivalent to G x E.
If no G x E is present, genotype effect is evaluated in the same design (equivalent to a paired t-test), intercept is equivalent to genotype effect.
If no G x E and no genotype effect are present, the environment (housing) effect is evaluated in all animals (n=53), without considering the littermate pairs.

#### Experimental Design

| Sex | Age (Months) | Environment | Genotype | Count |
| --- | --- | --- | --- | --- |
| Male | 2 | Iso | +/+ - +/- | 9 |
|  |  | Stand | +/+ - +/- | 9 |
|  |  | Enr | +/+ - +/- | 8 |

#### Test of gene-environment interaction

Analysis of Variance Table

Response: RCR
 Df Sum Sq Mean Sq F value Pr(>F)
Environment 2 0.06021 0.030107 0.3617 0.7004
Residuals 23 1.91422 0.083227

R-squared = 0.0305

#### Test of genotype main effect

Estimate Std. Error t value Pr(>|t|)
(Intercept) -0.0073 0.0551 -0.132 0.896

R-squared = 0

#### Experimental Design

| Sex | Age (Months) | Environment | Genotype | Count |
| --- | --- | --- | --- | --- |
| Male | 2 | Iso | +/+ | 9 |
|  |  |  | +/- | 9 |
|  |  | Stand | +/+ | 9 |
|  |  |  | +/- | 9 |
|  |  | Enr | +/+ | 9 |
|  |  |  | +/- | 8 |

#### Test of environment main effect

Analysis of Variance Table

Response: RCR
 Df Sum Sq Mean Sq F value Pr(>F)
Environment 2 0.0279 0.01396 0.0367 0.964
Residuals 50 19.0226 0.38045

R-squared = 0.00147

#### Overall mean in 2 months old male animals

Mean CI: [0.025, 0.975]
All 2.97 2.8 3.13

## RCR HC

G x E is evaluated in a paired design (difference between +/+ and +/- littermate pairs), environmental effect in this design is equivalent to G x E.
If no G x E is present, genotype effect is evaluated in the same design (equivalent to a paired t-test), intercept is equivalent to genotype effect.
If no G x E and no genotype effect are present, the environment (housing) effect is evaluated in all animals (n=53), without considering the littermate pairs.

#### Experimental Design

| Sex | Age (Months) | Environment | Genotype | Count |
| --- | --- | --- | --- | --- |
| Male | 2 | Iso | +/+ - +/- | 9 |
|  |  | Stand | +/+ - +/- | 9 |
|  |  | Enr | +/+ - +/- | 8 |

#### Test of gene-environment interaction

Analysis of Variance Table

Response: RCR
 Df Sum Sq Mean Sq F value Pr(>F)
Environment 2 0.6596 0.32981 0.7328 0.4914
Residuals 23 10.3511 0.45005

R-squared = 0.0599

#### Test of genotype main effect

Estimate Std. Error t value Pr(>|t|)
(Intercept) -0.0352 0.13 -0.27 0.789

R-squared = 0

#### Experimental Design

| Sex | Age (Months) | Environment | Genotype | Count |
| --- | --- | --- | --- | --- |
| Male | 2 | Iso | +/+ | 9 |
|  |  |  | +/- | 9 |
|  |  | Stand | +/+ | 9 |
|  |  |  | +/- | 9 |
|  |  | Enr | +/+ | 9 |
|  |  |  | +/- | 8 |

#### Test of environment main effect

Analysis of Variance Table

Response: RCR
 Df Sum Sq Mean Sq F value Pr(>F)
Environment 2 0.103 0.0517 0.0754 0.9275
Residuals 50 34.310 0.6862

R-squared = 0.003

#### Overall mean in 2 months old male animals

Mean CI: [0.025, 0.975]
All 3.42 3.19 3.64

## RCR Hemisphere, Age, Sex

### Brain hemisphere PFC

Interaction between genotype and hemisphere is evaluated in a double-paired design (difference between left and right hemisphere per animal, difference of this between +/+ and +/- littermate pairs), the intercept in this design is equivalent to gene-hemisphere interaction.
If no gene-hemisphere interaction is present, the hemisphere effect is evaluated in all animals (n=12) as the difference between left and right hemisphere, without considering the littermate pairs (equivalent to a paired t-test), intercept is equivalent to hemisphere effect.

#### Experimental Design

| Sex | Age (Months) | Environment | Genotype | Brain hemisphere | Count |
| --- | --- | --- | --- | --- | --- |
| Male | 2 | Stand | +/+ - +/- | Left - Right | 6 |

#### Test of gene-hemisphere interaction

Estimate Std. Error t value Pr(>|t|)
(Intercept) -0.253 0.206 -1.23 0.274

R-squared = 0

#### Experimental Design

| Sex | Age (Months) | Environment | Genotype | Brain hemisphere | Count |
| --- | --- | --- | --- | --- | --- |
| Male | 2 | Stand | +/+ | Left - Right | 6 |
|  |  |  | +/- | Left - Right | 6 |

#### Test of hemisphere main effect

Estimate Std. Error t value Pr(>|t|)
(Intercept) 0.124 0.0914 1.36 0.201

R-squared = 0

#### Overall mean RCR difference between left and right hemisphere

Mean CI: [0.025, 0.975]
All 0.12 -0.077 0.33

### Age PFC

Interaction between genotype and age is evaluated in a paired design (difference between +/+ and +/- littermate pairs), the age effect in this design is equivalent to gene-age interaction.
As a gene-age interaction is present, the genotype effect is evaluated separately in ten-month-old male littermate pairs (equivalent to a paired t-test), intercept is equivalent to genotype effect.

#### Experimental Design

| Sex | Age (Months) | Environment | Genotype | Count |
| --- | --- | --- | --- | --- |
| Male | 2 | Stand | +/+ - +/- | 15 |
|  | 10 |  | +/+ - +/- | 8 |

#### Test of gene-age interaction

Analysis of Variance Table

Response: RCR
 Df Sum Sq Mean Sq F value Pr(>F)
Age 1 1.7520 1.75198 30.348 1.821e-05
Residuals 21 1.2123 0.05773

R-squared = 0.591

#### Experimental Design

| Sex | Age (Months) | Environment | Genotype | Count |
| --- | --- | --- | --- | --- |
| Male | 10 | Stand | +/+ - +/- | 8 |

#### Test of genotype main effect in 10-month-old male animals

Estimate Std. Error t value Pr(>|t|)
(Intercept) 0.453 0.0723 6.27 0.000418

R-squared = 0

#### Group means in 10-month-old male animals

Mean CI: [0.025, 0.975]
+/- 4.35 4.13 4.58
+/+ 3.90 3.67 4.13

### Age HC

Interaction between genotype and age is evaluated in a paired design (difference between +/+ and +/- littermate pairs), the age effect in this design is equivalent to gene-age interaction.
The genotype effect is evaluated separately in ten-month-old male littermate pairs (equivalent to a paired t-test), intercept is equivalent to genotype effect.
If no G x A and no genotype effect are present, the age effect is evaluated in all animals (n=33), without considering the littermate pairs.

#### Experimental Design

| Sex | Age (Months) | Environment | Genotype | Count |
| --- | --- | --- | --- | --- |
| Male | 2 | Stand | +/+ - +/- | 9 |
|  | 10 |  | +/+ - +/- | 7 |

#### Test of gene-age interaction

Analysis of Variance Table

Response: RCR
 Df Sum Sq Mean Sq F value Pr(>F)
Age 1 0.1348 0.13484 0.2042 0.6583
Residuals 14 9.2464 0.66045

R-squared = 0.0144

#### Experimental Design

| Sex | Age (Months) | Environment | Genotype | Count |
| --- | --- | --- | --- | --- |
| Male | 10 | Stand | +/+ - +/- | 7 |

#### Test of genotype main effect in 10 months old male animals

Estimate Std. Error t value Pr(>|t|)
(Intercept) 0.225 0.215 1.04 0.336

R-squared = 0

#### Experimental Design

| Sex | Age (Months) | Environment | Genotype | Count |
| --- | --- | --- | --- | --- |
| Male | 2 | Stand | +/+ | 9 |
|  |  |  | +/- | 9 |
|  | 10 |  | +/+ | 7 |
|  |  |  | +/- | 8 |

#### Test of age main effect in standard housing

Analysis of Variance Table

Response: RCR
 Df Sum Sq Mean Sq F value Pr(>F)
Age 1 9.2801 9.2801 16.462 0.0003114
Residuals 31 17.4758 0.5637

R-squared = 0.347

#### Group means of 2- and 10-month-old male animals

Mean CI: [0.025, 0.975]
2-month 3.44 3.08 3.8
10-month 4.50 4.11 4.9

### Sex PFC

Interaction between genotype and sex is evaluated in a paired design (difference between +/+ and +/- littermate pairs), the sex effect in this design is equivalent to gene-sex interaction.
The genotype effect is evaluated separately in ten-month-old female littermate pairs (equivalent to a paired t-test), intercept is equivalent to genotype effect.
Sex effect is evaluated in all animals, irrespective of genotype.

#### Experimental Design

| Sex | Age (Months) | Environment | Genotype | Count |
| --- | --- | --- | --- | --- |
| Male | 10 | Stand | +/+ - +/- | 8 |
| Female |  |  | +/+ - +/- | 8 |

#### Test of gene-sex interaction

Analysis of Variance Table

Response: RCR
 Df Sum Sq Mean Sq F value Pr(>F)
Sex 1 2.3401 2.34013 4.1383 0.06132
Residuals 14 7.9167 0.56548

R-squared = 0.228

#### Experimental Design

| Sex | Age (Months) | Environment | Genotype | Count |
| --- | --- | --- | --- | --- |
| Female | 10 | Stand | +/+ - +/- | 8 |

#### Test of genotype main effect in 10-month-old female animals

Estimate Std. Error t value Pr(>|t|)
(Intercept) -0.312 0.369 -0.845 0.426

R-squared = 0

#### Experimental Design

| Sex | Age (Months) | Environment | Genotype | Count |
| --- | --- | --- | --- | --- |
| Male | 10 | Stand | +/+ | 8 |
|  |  |  | +/- | 8 |
| Female |  |  | +/+ | 8 |
|  |  |  | +/- | 8 |

#### Test of sex effect in 10 months old animals

Analysis of Variance Table

Response: RCR
 Df Sum Sq Mean Sq F value Pr(>F)
Sex 1 0.3018 0.30184 0.9524 0.3369
Residuals 30 9.5075 0.31692

R-squared = 0.0308

#### Overall mean in 10 months old female animals

Mean CI: [0.025, 0.975]
All 4.32 3.94 4.69

### Sex HC

Interaction between genotype and sex is evaluated in a paired design (difference between +/+ and +/- littermate pairs), the sex effect in this design is equivalent to gene-sex interaction.
The genotype effect is evaluated separately in ten-month-old female littermate pairs (equivalent to a paired t-test), intercept is equivalent to genotype effect.
Sex effect is evaluated in all animals, irrespective of genotype.

#### Experimental Design

| Sex | Age (Months) | Environment | Genotype | Count |
| --- | --- | --- | --- | --- |
| Male | 10 | Stand | +/+ - +/- | 7 |
| Female |  |  | +/+ - +/- | 7 |

#### Test of gene-sex interaction

Analysis of Variance Table

Response: RCR
 Df Sum Sq Mean Sq F value Pr(>F)
Sex 1 0.85616 0.85616 3.8069 0.07478
Residuals 12 2.69876 0.22490

R-squared = 0.241

#### Experimental Design

| Sex | Age (Months) | Environment | Genotype | Count |
| --- | --- | --- | --- | --- |
| Female | 10 | Stand | +/+ - +/- | 7 |

#### Test of genotype main effect in 10-month-old female animals

Estimate Std. Error t value Pr(>|t|)
(Intercept) -0.27 0.134 -2.01 0.0909

R-squared = 0

#### Experimental Design

| Sex | Age (Months) | Environment | Genotype | Count |
| --- | --- | --- | --- | --- |
| Male | 10 | Stand | +/+ | 7 |
|  |  |  | +/- | 8 |
| Female |  |  | +/+ | 7 |
|  |  |  | +/- | 7 |

#### Test of sex effect in 10 months old animals

Analysis of Variance Table

Response: RCR
 Df Sum Sq Mean Sq F value Pr(>F)
Sex 1 0.1618 0.16185 1.2641 0.2708
Residuals 27 3.4569 0.12804

R-squared = 0.0447

#### Overall mean in 10-month-old female animals

Mean CI: [0.025, 0.975]
All 4.35 4.21 4.49

## ETC complex protein levels PFC

For all five complexes, G x E is evaluated in a paired design (difference between +/+ and +/- littermate pairs), environmental effect in this design is equivalent to G x E.
If no G x E is present, genotype effect is evaluated in the same design (equivalent to a paired t-test), intercept is equivalent to genotype effect.
If no G x E and no genotype effect are present, the environment (housing) effect is evaluated in all animals (n=24), without considering the littermate pairs.

#### Experimental Design for test of gene-environment interaction and genotype main effect

| Sex | Age (Months) | Environment | Genotype | Count |
| --- | --- | --- | --- | --- |
| Male | 2 | Iso | +/+ - +/- | 4 |
|  |  | Stand | +/+ - +/- | 4 |
|  |  | Enr | +/+ - +/- | 4 |

#### Experimental Design for test of environment main effect

| Sex | Age (Months) | Environment | Genotype | Count |
| --- | --- | --- | --- | --- |
| Male | 2 | Iso | +/+ | 4 |
|  |  |  | +/- | 4 |
|  |  | Stand | +/+ | 4 |
|  |  |  | +/- | 4 |
|  |  | Enr | +/+ | 4 |
|  |  |  | +/- | 4 |

### Complex 1

#### Test of gene-environment interaction

Analysis of Variance Table

Response: Complex1
 Df Sum Sq Mean Sq F value Pr(>F)
Environment 2 0.05571 0.027857 0.1965 0.825
Residuals 9 1.27597 0.141774

R-squared = 0.0418

#### Test of genotype main effect

Estimate Std. Error t value Pr(>|t|)
(Intercept) -0.0407 0.1 -0.405 0.693

R-squared = 0

#### Test of environment main effect

Analysis of Variance Table

Response: Complex1
 Df Sum Sq Mean Sq F value Pr(>F)
Environment 2 0.28941 0.144705 2.3284 0.1221
Residuals 21 1.30512 0.062148

R-squared = 0.182

#### Overall mean

Mean CI: [0.025, 0.975]
All 0.88 0.769 0.992

### Complex 2

#### Test of gene-environment interaction

Analysis of Variance Table

Response: Complex2
 Df Sum Sq Mean Sq F value Pr(>F)
Environment 2 0.10758 0.053790 1.2051 0.3438
Residuals 9 0.40172 0.044635

R-squared = 0.211

#### Test of genotype main effect

Estimate Std. Error t value Pr(>|t|)
(Intercept) 0.0538 0.0621 0.867 0.405

R-squared = 0

#### Test of environment main effect

Analysis of Variance Table

Response: Complex2
 Df Sum Sq Mean Sq F value Pr(>F)
Environment 2 0.03213 0.016067 0.1708 0.8441
Residuals 21 1.97529 0.094061

R-squared = 0.016

#### Overall mean

Mean CI: [0.025, 0.975]
All 1.09 0.963 1.21

### Complex 3

#### Test of gene-environment interaction

Analysis of Variance Table

Response: Complex3
 Df Sum Sq Mean Sq F value Pr(>F)
Environment 2 0.048841 0.024421 0.7253 0.5105
Residuals 9 0.303032 0.033670

R-squared = 0.139

#### Test of genotype main effect

Estimate Std. Error t value Pr(>|t|)
(Intercept) -0.0824 0.0516 -1.6 0.139

R-squared = 0

#### Test of environment main effect

Analysis of Variance Table

Response: Complex3
 Df Sum Sq Mean Sq F value Pr(>F)
Environment 2 0.12674 0.063368 0.5249 0.5992
Residuals 21 2.53517 0.120722

R-squared = 0.0476

#### Overall mean

Mean CI: [0.025, 0.975]
All 0.916 0.772 1.06

### Complex 4

#### Test of gene-environment interaction

Analysis of Variance Table

Response: Complex4
 Df Sum Sq Mean Sq F value Pr(>F)
Environment 2 0.00769 0.003846 0.0662 0.9364
Residuals 9 0.52267 0.058074

R-squared = 0.0145

#### Test of genotype main effect

Estimate Std. Error t value Pr(>|t|)
(Intercept) 0.0241 0.0634 0.38 0.711

R-squared = 0

#### Test of environment main effect

Analysis of Variance Table

Response: Complex4
 Df Sum Sq Mean Sq F value Pr(>F)
Environment 2 0.03914 0.019568 0.3514 0.7078
Residuals 21 1.16932 0.055682

R-squared = 0.0324

#### Overall mean

Mean CI: [0.025, 0.975]
All 1 0.905 1.1

### Complex 5

#### Test of gene-environment interaction

Analysis of Variance Table

Response: Complex5
 Df Sum Sq Mean Sq F value Pr(>F)
Environment 2 0.16416 0.082079 2.3748 0.1485
Residuals 9 0.31107 0.034563

R-squared = 0.345

#### Test of genotype main effect

Estimate Std. Error t value Pr(>|t|)
(Intercept) 9.43e-05 0.06 0.00157 0.999

R-squared = 0

#### Test of environment main effect

Analysis of Variance Table

Response: Complex5
 Df Sum Sq Mean Sq F value Pr(>F)
Environment 2 0.05401 0.027007 0.5209 0.6015
Residuals 21 1.08880 0.051848

R-squared = 0.0473

#### Overall mean

Mean CI: [0.025, 0.975]
All 0.98 0.886 1.07

## ETC complex protein levels HC

For all five complexes, G x E is evaluated in a paired design (difference between +/+ and +/- littermate pairs), environmental effect in this design is equivalent to G x E.
If no G x E is present, genotype effect is evaluated in the same design (equivalent to a paired t-test), intercept is equivalent to genotype effect.
If no G x E and no genotype effect are present, the environment (housing) effect is evaluated in all animals (n=24), without considering the littermate pairs.

#### Experimental Design for test of gene-environment interaction and genotype main effect

| Sex | Age (Months) | Environment | Genotype | Count |
| --- | --- | --- | --- | --- |
| Male | 2 | Iso | +/+ - +/- | 4 |
|  |  | Stand | +/+ - +/- | 4 |
|  |  | Enr | +/+ - +/- | 4 |

#### Experimental Design for test of environment main effect

| Sex | Age (Months) | Environment | Genotype | Count |
| --- | --- | --- | --- | --- |
| Male | 2 | Iso | +/+ | 4 |
|  |  |  | +/- | 4 |
|  |  | Stand | +/+ | 4 |
|  |  |  | +/- | 4 |
|  |  | Enr | +/+ | 4 |
|  |  |  | +/- | 4 |

### Complex 1

#### Test of gene-environment interaction

Analysis of Variance Table

Response: Complex1
 Df Sum Sq Mean Sq F value Pr(>F)
Environment 2 0.04923 0.024614 0.5384 0.6014
Residuals 9 0.41149 0.045721

R-squared = 0.107

#### Test of genotype main effect

Estimate Std. Error t value Pr(>|t|)
(Intercept) 0.0357 0.0591 0.604 0.558

R-squared = 0

#### Test of environment main effect

Analysis of Variance Table

Response: Complex1
 Df Sum Sq Mean Sq F value Pr(>F)
Environment 2 0.21154 0.10577 0.9252 0.412
Residuals 21 2.40076 0.11432

R-squared = 0.081

#### Overall mean

Mean CI: [0.025, 0.975]
All 1.04 0.893 1.18

### Complex 2

#### Test of gene-environment interaction

Analysis of Variance Table

Response: Complex2
 Df Sum Sq Mean Sq F value Pr(>F)
Environment 2 0.18209 0.091046 2.1023 0.1782
Residuals 9 0.38978 0.043308

R-squared = 0.318

#### Test of genotype main effect

Estimate Std. Error t value Pr(>|t|)
(Intercept) 0.0172 0.0658 0.261 0.799

R-squared = 0

#### Test of environment main effect

Analysis of Variance Table

Response: Complex2
 Df Sum Sq Mean Sq F value Pr(>F)
Environment 2 0.1846 0.092301 0.8949 0.4237
Residuals 21 2.1660 0.103141

R-squared = 0.0785

#### Overall mean

Mean CI: [0.025, 0.975]
All 0.936 0.801 1.07

### Complex 3

#### Test of gene-environment interaction

Analysis of Variance Table

Response: Complex3
 Df Sum Sq Mean Sq F value Pr(>F)
Environment 2 0.057584 0.028792 1.0436 0.3912
Residuals 9 0.248309 0.027590

R-squared = 0.188

#### Test of genotype main effect

Estimate Std. Error t value Pr(>|t|)
(Intercept) -0.0323 0.0481 -0.671 0.516

R-squared = 0

#### Test of environment main effect

Analysis of Variance Table

Response: Complex3
 Df Sum Sq Mean Sq F value Pr(>F)
Environment 2 0.08392 0.041959 0.3587 0.7028
Residuals 21 2.45622 0.116963

R-squared = 0.033

#### Overall mean

Mean CI: [0.025, 0.975]
All 0.986 0.846 1.13

### Complex 4

#### Test of gene-environment interaction

Analysis of Variance Table

Response: Complex4
 Df Sum Sq Mean Sq F value Pr(>F)
Environment 2 0.070817 0.035408 1.496 0.2748
Residuals 9 0.213011 0.023668

R-squared = 0.25

#### Test of genotype main effect

Estimate Std. Error t value Pr(>|t|)
(Intercept) 0.009 0.0464 0.194 0.85

R-squared = 0

#### Test of environment main effect

Analysis of Variance Table

Response: Complex4
 Df Sum Sq Mean Sq F value Pr(>F)
Environment 2 0.06758 0.033789 0.369 0.6958
Residuals 21 1.92303 0.091573

R-squared = 0.0339

#### Overall mean

Mean CI: [0.025, 0.975]
All 0.974 0.85 1.1

### Complex 5

#### Test of gene-environment interaction

Analysis of Variance Table

Response: Complex5
 Df Sum Sq Mean Sq F value Pr(>F)
Environment 2 0.06563 0.032814 0.4214 0.6685
Residuals 9 0.70089 0.077877

R-squared = 0.0856

#### Test of genotype main effect

Estimate Std. Error t value Pr(>|t|)
(Intercept) 0.0673 0.0762 0.883 0.396

R-squared = 0

#### Test of environment main effect

Analysis of Variance Table

Response: Complex5
 Df Sum Sq Mean Sq F value Pr(>F)
Environment 2 0.20195 0.10097 0.9397 0.4066
Residuals 21 2.25652 0.10745

R-squared = 0.0821

#### Overall mean

Mean CI: [0.025, 0.975]
All 0.938 0.799 1.08

## MitoSOX PFC

G x E is evaluated in a paired design (difference between +/+ and +/- littermate pairs), environmental effect in this design is equivalent to G x E.
If no G x E is present, genotype effect is evaluated in the same design (equivalent to a paired t-test), intercept is equivalent to genotype effect.
If no G x E and no genotype effect are present, the environment (housing) effect is evaluated in all animals (n=53), without considering the littermate pairs.
In the final model, isolation and standard housing were combined into one group, as they had almost identical mean values.
Since variance heteroscedasticity was present, the models were estimated by weighed least squares (WLS) with weights set to the reciprocal of the OLS-residual variance.

#### Experimental Design

| Sex | Age (Months) | Environment | Genotype | Count |
| --- | --- | --- | --- | --- |
| Male | 2 | Iso | +/+ - +/- | 9 |
|  |  | Stand | +/+ - +/- | 9 |
|  |  | Enr | +/+ - +/- | 8 |

#### Test of gene-environment interaction

Analysis of Variance Table

Response: MitoSOX
 Df Sum Sq Mean Sq F value Pr(>F)
Environment 2 0.985 0.49255 0.2643 0.77
Residuals 23 42.863 1.86359

R-squared = 0.0225

#### Test of genotype main effect

Estimate Std. Error t value Pr(>|t|)
(Intercept) -0.625 0.613 -1.02 0.318

R-squared = 0

#### Experimental Design for test of environment main effect

| Sex | Age (Months) | Environment | Genotype | Count |
| --- | --- | --- | --- | --- |
| Male | 2 | Iso | +/+ | 9 |
|  |  |  | +/- | 9 |
|  |  | Stand | +/+ | 9 |
|  |  |  | +/- | 9 |
|  |  | Enr | +/+ | 9 |
|  |  |  | +/- | 8 |

#### Test of environment main effect

Analysis of Variance Table

Response: MitoSOX
 Df Sum Sq Mean Sq F value Pr(>F)
Environment 2 20.596 10.2979 5.6446 0.006161
Residuals 50 91.219 1.8244

R-squared = 0.184

#### Final model

Estimate Std. Error t value Pr(>|t|)
(Intercept) 10.85 1.42 7.65 5.12e-10
Env2ISO/STAND -4.58 1.46 -3.13 2.89e-03

#### Group means

Mean CI: [0.025, 0.975]
Enriched 10.85 8.004 13.699
Isolation/Standard 6.27 5.542 6.997

## MitoSOX HC

G x E is evaluated in a paired design (difference between +/+ and +/- littermate pairs), environmental effect in this design is equivalent to G x E.
If no G x E is present, genotype effect is evaluated in the same design (equivalent to a paired t-test), intercept is equivalent to genotype effect.
If no G x E and no genotype effect are present, the environment (housing) effect is evaluated in all animals (n=53), without considering the littermate pairs.
Since variance heteroscedasticity was present, the models were estimated by weighed least squares (WLS) with weights set to the reciprocal of the OLS-residual variance.

#### Experimental Design

| Sex | Age (Months) | Environment | Genotype | Count |
| --- | --- | --- | --- | --- |
| Male | 2 | Iso | +/+ - +/- | 9 |
|  |  | Stand | +/+ - +/- | 9 |
|  |  | Enr | +/+ - +/- | 8 |

#### Test of gene-environment interaction

Analysis of Variance Table

Response: MitoSOX
 Df Sum Sq Mean Sq F value Pr(>F)
Environment 2 0.273 0.13665 0.0525 0.949
Residuals 23 59.848 2.60208

R-squared = 0.00455

#### Test of genotype main effect

Estimate Std. Error t value Pr(>|t|)
(Intercept) 0.153 1.27 0.12 0.905

R-squared = 0

#### Experimental Design for test of environment main effect

| Sex | Age (Months) | Environment | Genotype | Count |
| --- | --- | --- | --- | --- |
| Male | 2 | Iso | +/+ | 9 |
|  |  |  | +/- | 9 |
|  |  | Stand | +/+ | 9 |
|  |  |  | +/- | 9 |
|  |  | Enr | +/+ | 9 |
|  |  |  | +/- | 8 |

#### Test of environment main effect

Analysis of Variance Table

Response: MitoSOX
 Df Sum Sq Mean Sq F value Pr(>F)
Environment 2 6.731 3.3657 1.4008 0.2559
Residuals 50 120.140 2.4028

R-squared = 0.0531

#### Overall mean

Mean CI: [0.025, 0.975]
All 7.48 6.46 8.49

## TMRE PFC

G x E is evaluated in a paired design (difference between +/+ and +/- littermate pairs), environmental effect in this design is equivalent to G x E.
If no G x E is present, genotype effect is evaluated in the same design (equivalent to a paired t-test), intercept is equivalent to genotype effect.
If no G x E and no genotype effect are present, the environment (housing) effect is evaluated in all animals (n=53), without considering the littermate pairs.
Since variance heteroscedasticity was present, the models were estimated by weighed least squares (WLS) with weights set to the reciprocal of the OLS-residual variance.

#### Experimental Design

| Sex | Age (Months) | Environment | Genotype | Count |
| --- | --- | --- | --- | --- |
| Male | 2 | Iso | +/+ - +/- | 9 |
|  |  | Stand | +/+ - +/- | 9 |
|  |  | Enr | +/+ - +/- | 8 |

#### Test of gene-environment interaction

Analysis of Variance Table

Response: TMRE
 Df Sum Sq Mean Sq F value Pr(>F)
Environment 2 1.429 0.7144 0.3626 0.6998
Residuals 23 45.314 1.9702

R-squared = 0.0306

#### Test of genotype main effect

Estimate Std. Error t value Pr(>|t|)
(Intercept) -2.44 1.29 -1.9 0.0696

R-squared = 0

#### Experimental Design for test of environment main effect

| Sex | Age (Months) | Environment | Genotype | Count |
| --- | --- | --- | --- | --- |
| Male | 2 | Iso | +/+ | 9 |
|  |  |  | +/- | 9 |
|  |  | Stand | +/+ | 9 |
|  |  |  | +/- | 9 |
|  |  | Enr | +/+ | 9 |
|  |  |  | +/- | 8 |

#### Test of environment main effect

Analysis of Variance Table

Response: TMRE
 Df Sum Sq Mean Sq F value Pr(>F)
Environment 2 2.076 1.0381 0.6175 0.5434
Residuals 50 84.058 1.6812

R-squared = 0.0241

#### Overall mean

Mean CI: [0.025, 0.975]
All 17.2 15.8 18.5

## TMRE HC

G x E is evaluated in a paired design (difference between +/+ and +/- littermate pairs), environmental effect in this design is equivalent to G x E.
If no G x E is present, genotype effect is evaluated in the same design (equivalent to a paired t-test), intercept is equivalent to genotype effect.
If no G x E and no genotype effect are present, the environment (housing) effect is evaluated in all animals (n=53), without considering the littermate pairs.
Since variance heteroscedasticity was present, the models were estimated by weighed least squares (WLS) with weights set to the reciprocal of the OLS-residual variance.

#### Experimental Design

| Sex | Age (Months) | Environment | Genotype | Count |
| --- | --- | --- | --- | --- |
| Male | 2 | Iso | +/+ - +/- | 9 |
|  |  | Stand | +/+ - +/- | 9 |
|  |  | Enr | +/+ - +/- | 8 |

#### Test of gene-environment interaction

Analysis of Variance Table

Response: TMRE
 Df Sum Sq Mean Sq F value Pr(>F)
Environment 2 2.971 1.4856 0.7234 0.4958
Residuals 23 47.236 2.0537

R-squared = 0.0592

#### Test of genotype main effect

Estimate Std. Error t value Pr(>|t|)
(Intercept) -2.48 1.19 -2.09 0.0471

R-squared = 0

#### Experimental Design for test of environment main effect

| Sex | Age (Months) | Environment | Genotype | Count |
| --- | --- | --- | --- | --- |
| Male | 2 | Iso | +/+ | 9 |
|  |  |  | +/- | 9 |
|  |  | Stand | +/+ | 9 |
|  |  |  | +/- | 9 |
|  |  | Enr | +/+ | 9 |
|  |  |  | +/- | 8 |

#### Test of environment main effect

Analysis of Variance Table

Response: TMRE
 Df Sum Sq Mean Sq F value Pr(>F)
Environment 2 2.483 1.2414 0.6976 0.5026
Residuals 50 88.981 1.7796

R-squared = 0.0271

#### Overall mean

Mean CI: [0.025, 0.975]
All 18.6 16.9 20.3

**Supplementary Figures**


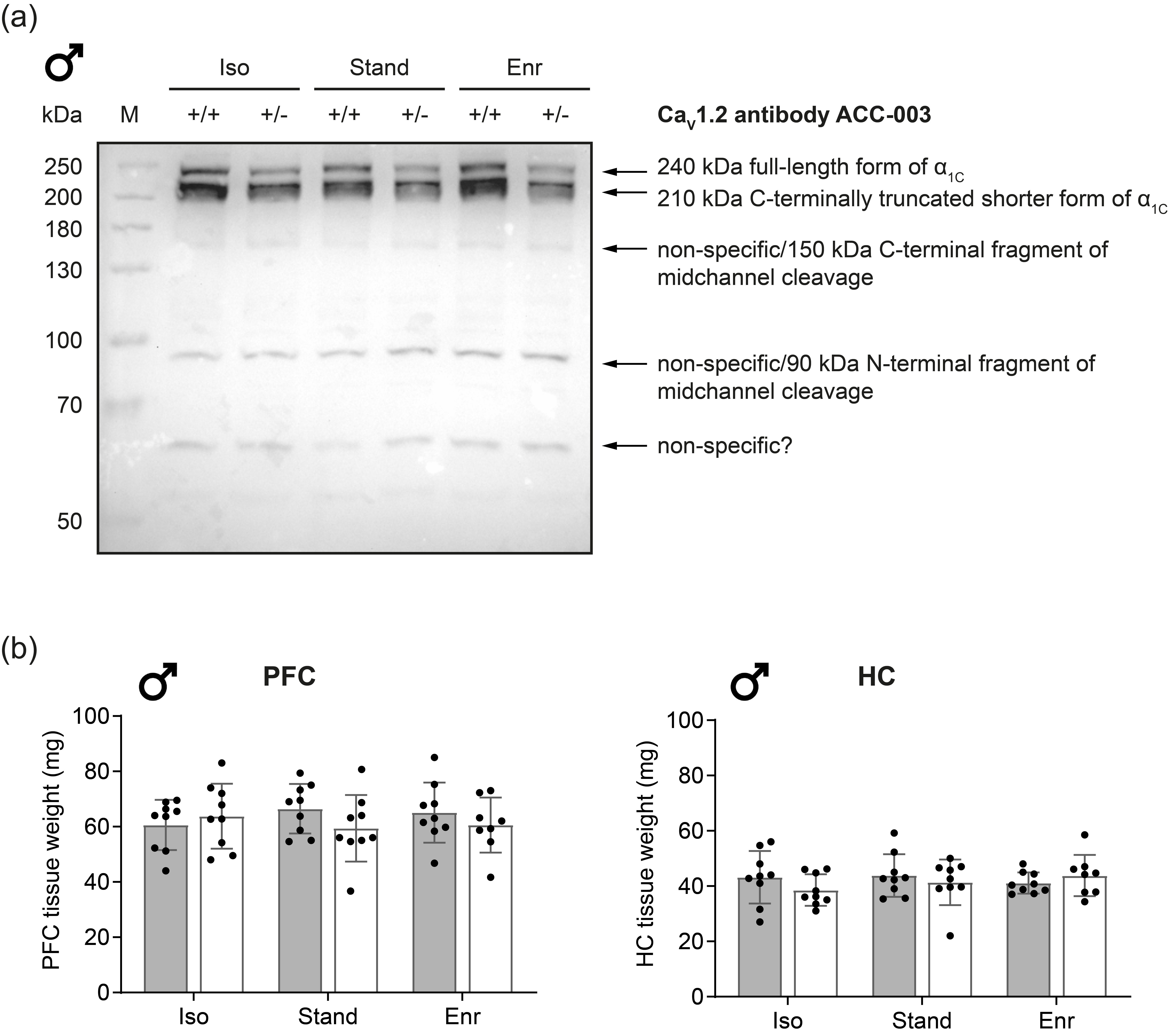


**Supplementary Figure S1.** Representative whole immunoblot with Ca_V_1.2 antibody and PFC/HC tissue weight

(a) This exemplary whole blot was labeled with the Ca_V_1.2 antibody ACC-003 (Alomone Labs, Jerusalem, Israel) illustrating the long (240 kDa) and short (210 kDa) form of the α_1C_-subunit of Ca_V_1.2. (b) PFC and HC tissue weight was determined immediately after extraction (mean ± SD, n = 8-9). PFC, prefrontal cortex; HC, hippocampus; +/+, wildtype *Cacna1c^+/+^* (grey bars); +/-, heterozygous *Cacna1c^+/-^* (clear bars); Iso, isolation; Stand, standard housing; Enr, enrichment; M, marker.


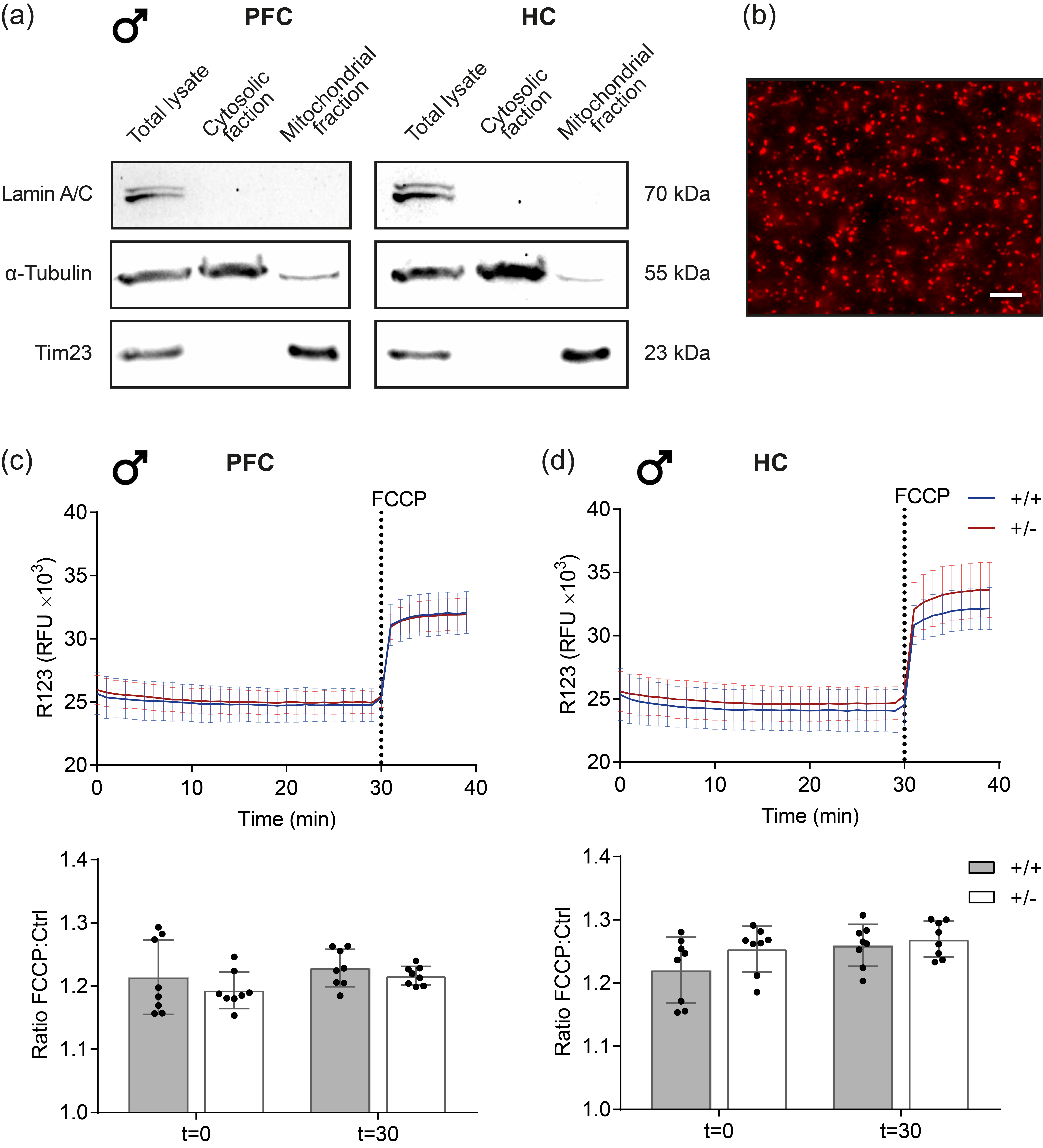


**Supplementary Figure S2.** Quality of the isolated mitochondria from rat brain tissue

(a) The fractionation quality was evaluated by Western blot using the total lysate, the cytosolic and the mitochondrial fraction of PFC and HC brain tissue. Lamin A/C was used as nuclear and α-Tubulin as cytosolic marker. The inner mitochondrial membrane protein Tim 23 served as indicator for mitochondrial enrichment. (b) To visualize mitochondria, the enriched mitochondrial fraction was stained with MitoTracker Deep Red. One representative image is shown. Scale bar 10 μm. Mitochondria from (c) the right PFC and (d) the right HC of male *Cacna1c^+/+^*-Stand and *Cacna1c^+/-^*-Stand rats were stained with rhodamine 123 (R123) to test their constant integrity after the isolation procedure. Positively charged R123 accumulates in mitochondria with intact membrane potential (ΔΨ_m_) leading to a time stable fluorescence quenching over 30 min. This is also evident from the constant FCCP to control ratios after zero and 30 min (t = 0, t = 30) (PFC, p = 0.0235; HC, p = 0.0117; paired t-test). The injection of FCCP (0.5 µM) as internal control dissipates the ΔΨ_m_ resulting in an increase in R123 fluorescence. Mean ± SD, n = 8; PFC, prefrontal cortex; HC, hippocampus; RFU, relative fluorescence units; +/+, wildtype *Cacna1c^+/+^* (grey bars); +/-, heterozygous *Cacna1c^+/-^* (clear bars).

**
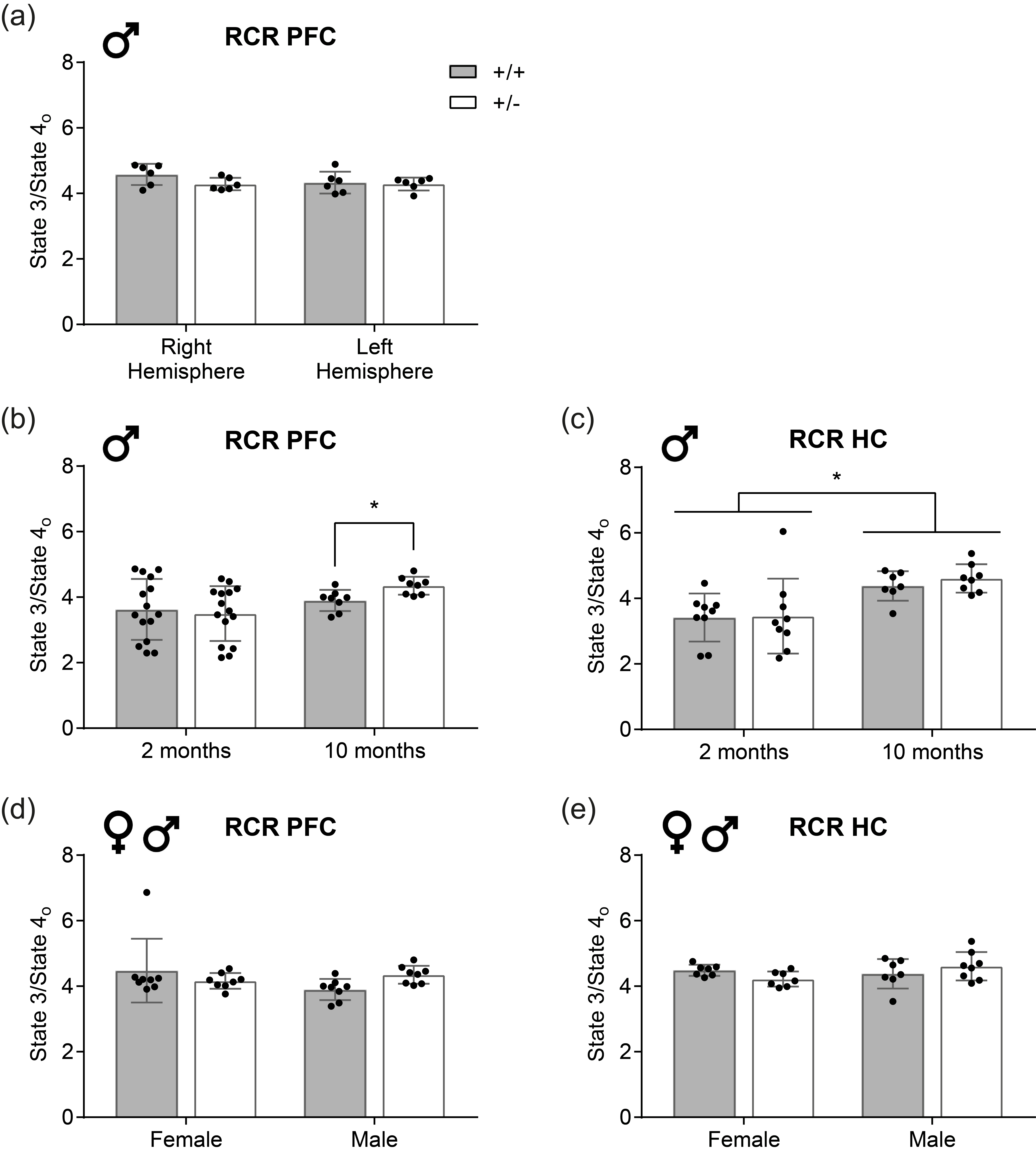
**

**Supplementary Figure S3.** Respiratory control ratio of mitochondria isolated from rats with differing age, sex and from both brain hemispheres

Respiratory control ratio (RCR) calculated from the OCR measurements using isolated mitochondria from: (a) right and left prefrontal cortex of two-month-old male *Cacna1c^+/+^*-Stand and *Cacna1c^+/-^*-Stand rats, (b) the right prefrontal cortex and (c) the right hippocampus of two- versus ten-month-old male *Cacna1c^+/-^* rats and *Cacna1c^+/+^* littermate controls which were kept under standard housing conditions, (d) the right prefrontal cortex and (e) the right hippocampus of ten-month-old male and female *Cacna1c^+/+^*-Stand and *Cacna1c^+/-^*-Stand rats (mean ± SD, pooled data). Statistical significance is highlighted by an asterisk (*). +/+, wildtype *Cacna1c^+/+^* (grey bars); +/-, heterozygous *Cacna1c^+/-^* (clear bars).


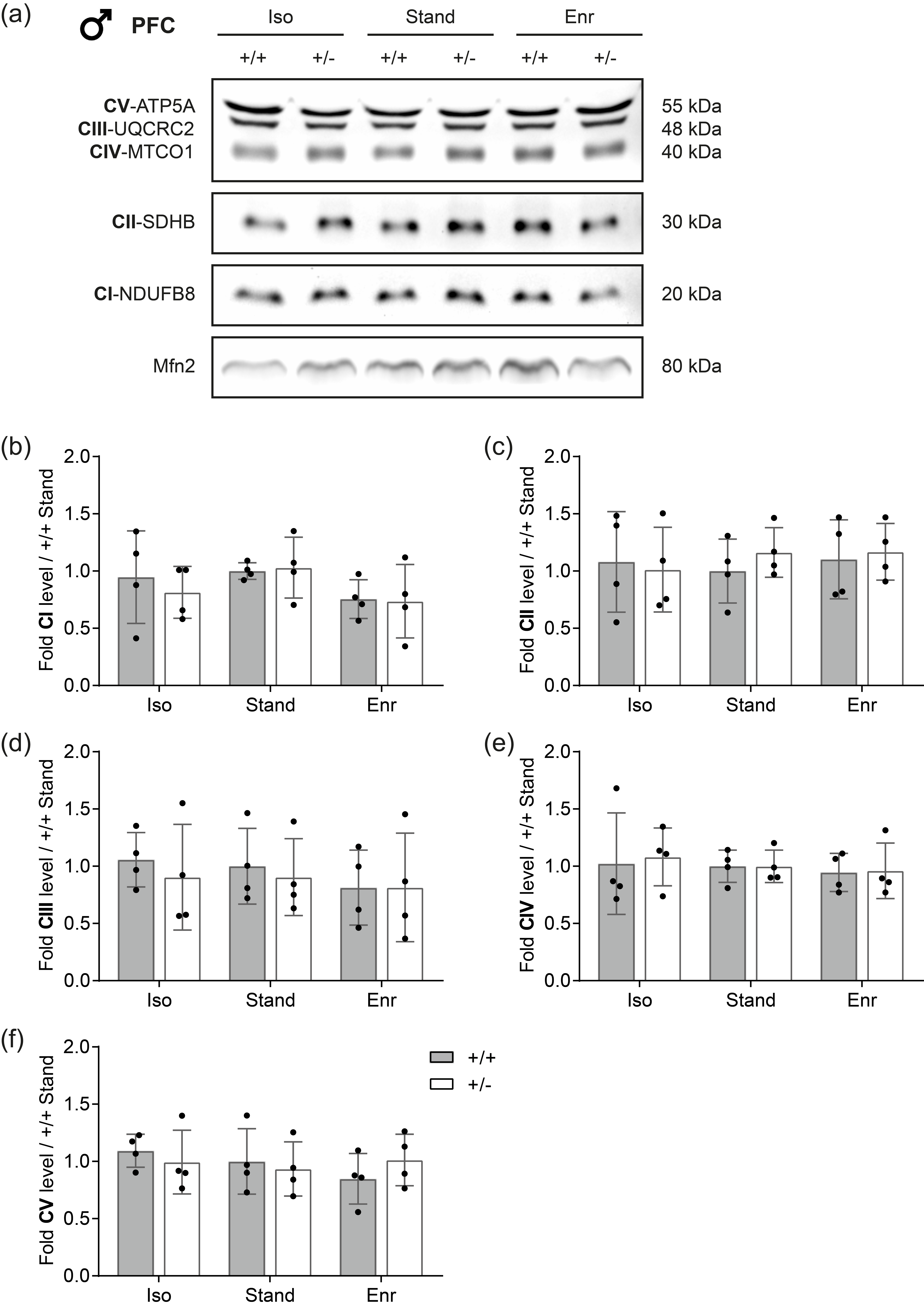


**Supplementary Figure S4.** Protein levels of the five electron transport chain complexes in right prefrontal cortex

Relative protein levels of the five electron transport chain complexes (CI-CV) were assessed in isolated PFC mitochondria from two-month-old male rats. (a) One representative immunoblot and (b-f) the densitometric quantification of the individual complexes (CI-CV) are depicted. The values are normalized to the mitochondrial outer membrane protein mitofusin 2 (Mfn2) and presented as fold of *Cacna1c^+/+^*-Stand (mean ± SD, n = 4). +/+, wildtype *Cacna1c^+/+^* (grey bars); +/-, heterozygous *Cacna1c^+/-^* (clear bars); Iso, isolation; Stand, standard housing; Enr, enrichment.


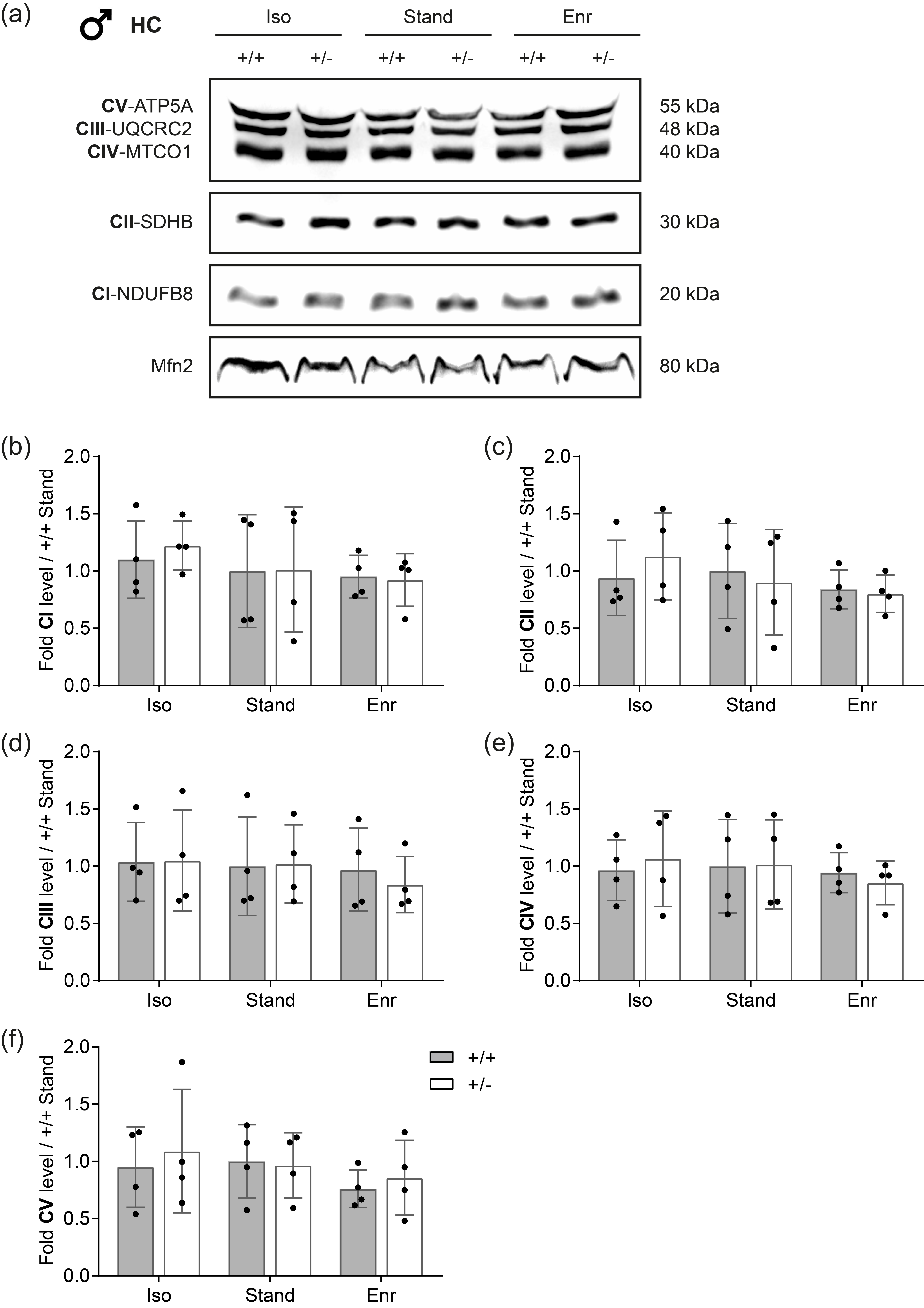


**Supplementary Figure S5.** Protein levels of the five electron transport chain complexes in right hippocampus

Relative protein levels of the five electron transport chain complexes (CI-CV) were assessed in isolated HC mitochondria from two-month-old male rats. (a) One representative immunoblot and (b-f) the densitometric quantification of the individual complexes (CI-CV) are depicted. The values are normalized to the mitochondrial outer membrane protein mitofusin 2 (Mfn2) and presented as fold of *Cacna1c^+/+^*-Stand (mean ± SD, n = 4). +/+, wildtype *Cacna1c^+/+^* (grey bars); +/-, heterozygous *Cacna1c^+/-^* (clear bars); Iso, isolation; Stand, standard housing; Enr, enrichment.


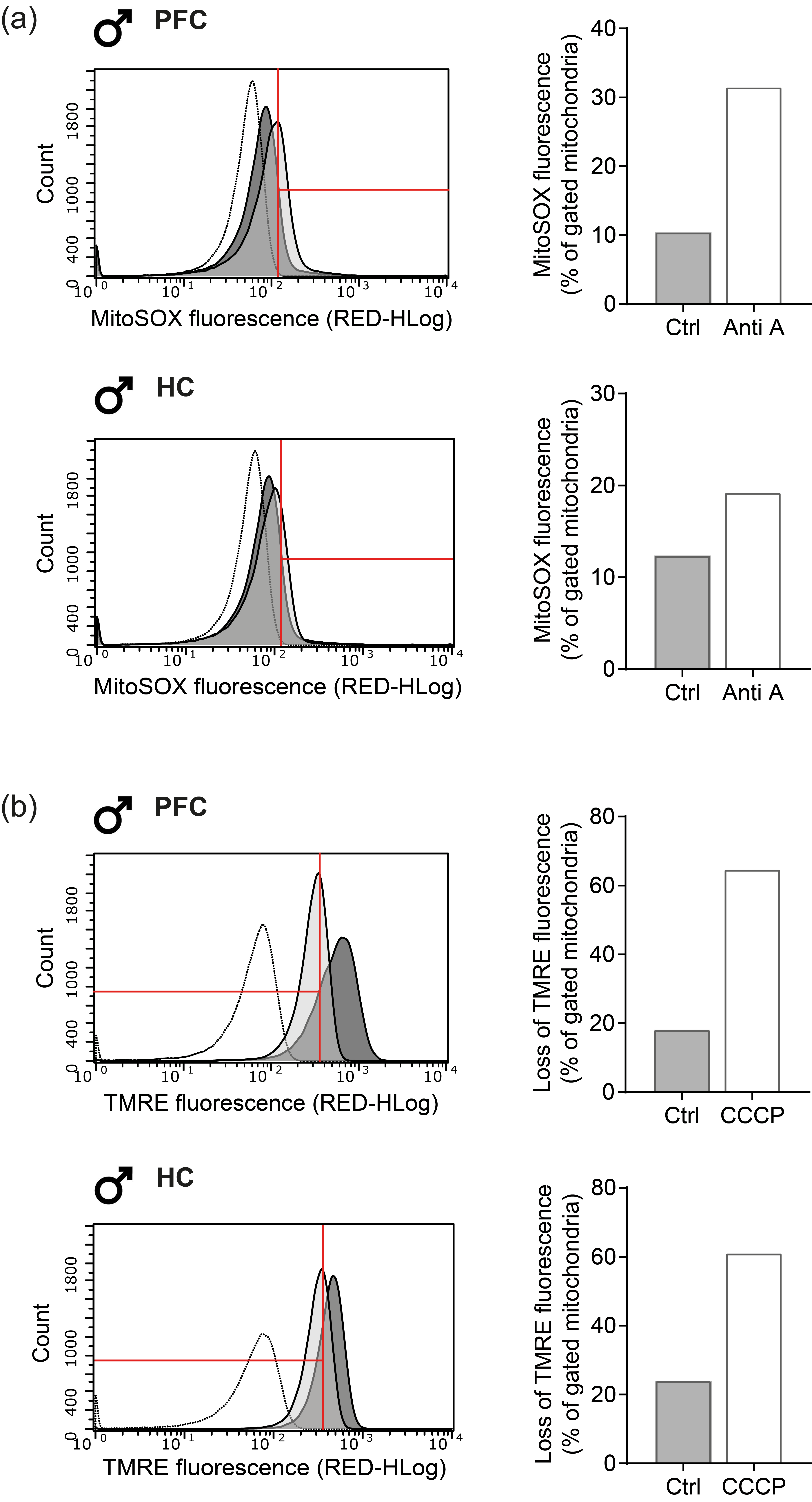


**Supplementary Figure S6.** Exemplary histograms and quantifications of flow cytometric measurements with isolated mitochondria from rat PFC and HC

Freshly isolated mitochondria were stained with (a) MitoSOX (1.25 μM) and (b) TMRE (0.2 μM). Antimycin A (Anti A, 10 μM) and CCCP (50 µM) respectively were used as validating positive control. The complex III inhibitor antimycin A increased the formation of superoxide leading to a rightward shift in MitoSOX fluorescence. Incubation of mitochondria with CCCP resulted in a decrease in membrane potential, which is visible as a left shift of the peak, indicating a loss of TMRE fluorescence. The histograms (left panel) illustrate that the gate (red) was set at the inflection point of the reference graph (*Cacna1c^+/+^*-Stand, dark grey). The clear graphs with the dotted line represent the unstained controls. The dark grey graphs constitute the control samples, the light grey graphs the Anti A/CCCP-treated samples, and the grey areas the overlay of both graphs. The bar graphs (right panel) display the percentage of mitochondria within the gate (n = 1). PFC, prefrontal cortex; HC, hippocampus.
